# Population-Based Discrimination of Classical and Few-Photon Microwave Fields with a Flux-Tunable Transmon Qudit

**DOI:** 10.3390/s26206538

**Published:** 2026-10-15

**Authors:** Andrey V. Pashchenko, Olga V. Tikhonova, Irina A. Solovykh, Igor I. Soloviev, Maxim V. Tereshonok, Nikolay V. Klenov, Dmitry S. Yakovlev

**Affiliations:** 1 Science and Research Department, Moscow Technical University of Communication and Informatics (MTUCI), 111024 Moscow, Russia; paschenko.andrey2014@yandex.ru (A.V.P.); m.v.tereshonok@mtuci.ru (M.V.T.); 2 Faculty of Physics, Lomonosov Moscow State University, 119991 Moscow, Russia; ovtikhonova@physics.msu.ru (O.V.T.); tereshchenko.ia19@physics.msu.ru (I.A.S.); 3 Skobeltsyn Institute of Nuclear Physics, Lomonosov Moscow State University, 119991 Moscow, Russia; i.i.soloviev@pn.sinp.msu.ru; 4 Kotel’nikov Institute of Radio Engineering and Electronics of RAS, 125009 Moscow, Russia; 5 Laboratoire de Physique et d’Etude des Matériaux, ESPCI-Paris, PSL Research University, 75005 Paris, France; dimitry.yakovlev@espci.fr

**Keywords:** transmon qudit, circuit quantum electrodynamics, microwave photon detection, nonclassical states, photon statistics, Fock states, coherent states, quantum sensing, two-tone spectroscopy, Ramsey interferometry

## Abstract

Interactions between few-photon microwave fields and superconducting artificial atoms provide a method to study photon statistics and manipulate quantum states for photonic quantum information processing. Superconducting transmons couple to microwave fields through an anharmonic ladder of energy levels, producing a response beyond the two-level description. Here, we propose and analyze a detector based on a flux-tunable transmon qudit in circuit quantum electrodynamics. Within a Kerr-type model, we calculate its population response to classical-field excitation and quantized Fock and coherent states. In the four-photon example, a selected detuning produces periodic, nearly complete transfer to the fourth transmon level for a Fock input, while a coherent input of equal mean energy gives a population of approximately 0.2 at the same interaction time. In the linear regime, we derive a target-population bound for classical inputs. From single-tone and two-tone spectroscopy, analyzed using the cosine transmon Hamiltonian, we extract circuit parameters and resolve higher transitions. Ramsey interferometry characterizes dephasing on the fundamental transition. We propose a detection protocol combining flux-selected interaction with dispersive population readout. Our results establish a population-based framework for microwave-state discrimination using a superconducting artificial atom.

## 1. Introduction

Nonclassical microwave states of the electromagnetic field are a key resource for photonic quantum information processing [1,2,3,4,5]. In superconducting circuit quantum electrodynamics (cQED) [6,7], this resource perspective is no longer purely conceptual: Fock states, engineered superpositions [8], and strongly nonlinear cavity states can now be generated and controlled with high precision in resonator–qubit platforms [9,10,11,12,13,14,15,16,17,18,19].

The remaining challenge extends beyond state preparation to efficient state verification. Full quadrature-based characterization by homodyne or heterodyne techniques is extremely powerful, yet it typically requires a relatively elaborate measurement chain and is most natural when the goal is complete state tomography [20,21].

A conceptually different approach is to use the nonlinear quantum system already coupled to the field as the detector itself [22,23,24,25,26,27,28]. In this context, the transmon is especially attractive. Originally introduced as a charge-noise-protected superconducting artificial atom, it combines long coherence with sufficiently weak anharmonicity to make several neighboring transitions experimentally accessible [29,30,31]. When operated intentionally beyond the two-level approximation, the transmon becomes a qudit whose multilevel dynamics carry significantly more information than the binary response of an idealized qubit [32,33,34]. This idea is supported by previous experiments in which transmon qudits were used as on-chip microwave sensors, as well as by recent demonstrations of coherent control and readout of many transmon levels [35,36,37].

Our previous work developed a Kerr-type description of a transmon qudit interacting with a few-photon field and identified detuning conditions for selective population transfer [38]. That study addressed control of the qudit by the field. Here, we examine the same interaction from a detection perspective: the field is the input to be distinguished, and the transmon level-population vector is the detector response. More recent theoretical and experimental studies of wave mixing and multiphoton scattering on superconducting two-level systems have sharpened this picture, showing that classical and nonclassical microwave fields can generate measurably different spectral responses and can even reveal sub-Poissonian photon statistics directly [39,40,41].

We compare the populations generated by Fock and coherent resonator inputs with equal mean energy and calculate the response to prescribed classical-field excitation as a separate reference. The central observable is $W\left(t\right)=(W_{0}\left(t\right),W_{1}\left(t\right),\ldots )$, evaluated at a common interaction time. In the illustrative nonlinear model, a selected detuning produces nearly complete transfer from a four-photon Fock input to the fourth transmon level, whereas the corresponding coherent input produces a substantially smaller target population. We examine the resulting population distributions as signatures for discriminating these specified inputs. In the linear regime, we also derive a target-population bound valid for classical input states.

**Figure 1:**
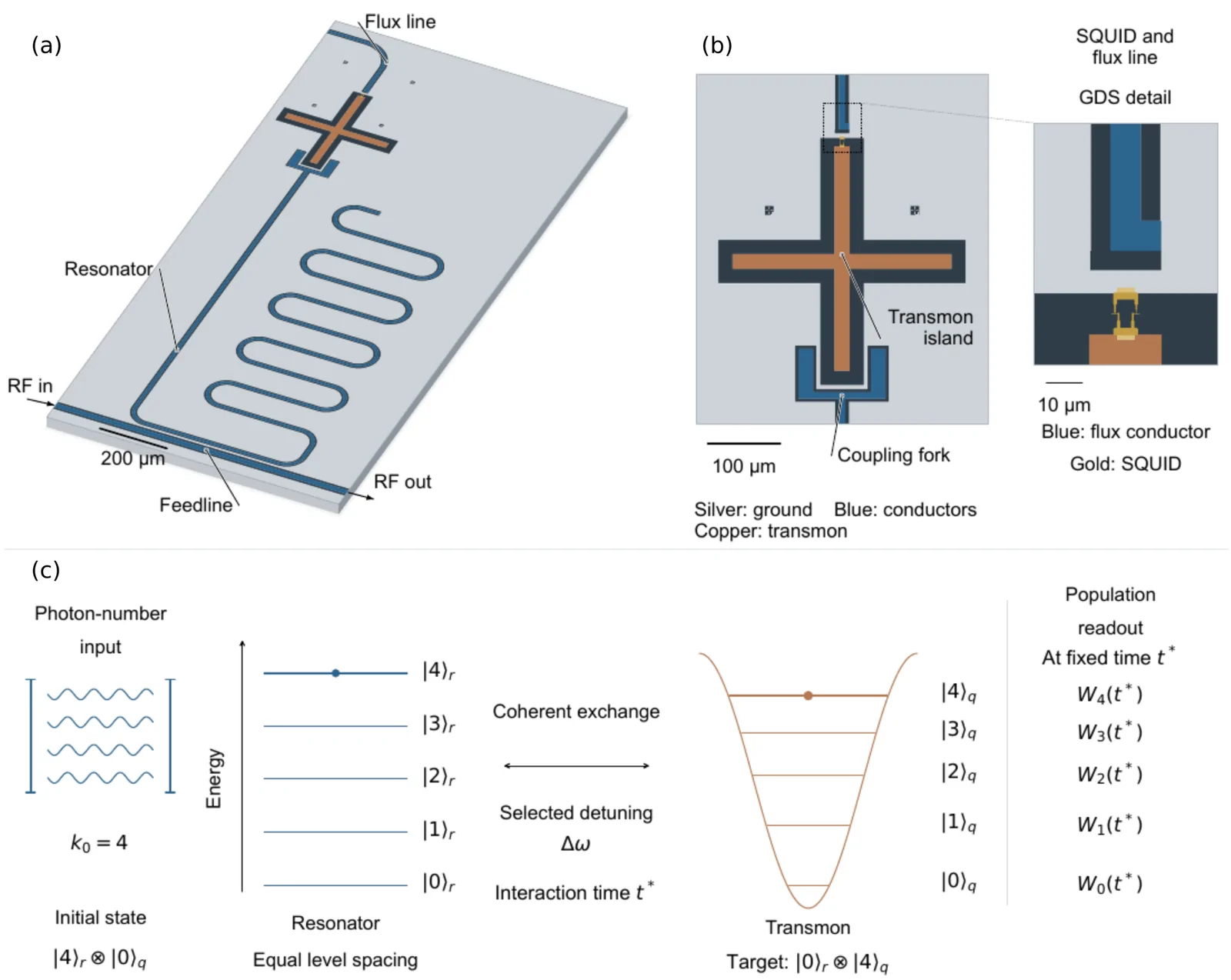
Detector concept. (**a**) Schematic resonator–transmon device. The coplanar resonator is coupled to the feedline and to the transmon. A local flux line controls the transmon frequency. (**b**) Zoomed coupling and flux-control region, with an inset showing the SQUID and flux-line geometry. (**c**) Proposed four-photon protocol. The resonator starts in ${|4\rangle }_{r}$ and the qudit in ${|0\rangle }_{q}$. Excitation exchange at a selected detuning generates the qudit populations evaluated at a fixed interaction time $t^{*}$. Levels 0–4 are shown for this Fock-input example.

We complement this analysis with single-tone and two-tone spectroscopy and Ramsey measurements of transmon–resonator devices. Fitting the spectra with the cosine transmon Hamiltonian determines circuit parameters and the resolved level structure, and Ramsey interferometry characterizes coherence on the fundamental transition. These measurements provide spectral and coherence scales relevant to the proposed detector. We formulate a two-stage protocol in which the qudit interacts with the signal resonator at a selected flux bias and is subsequently brought to a dispersive readout point, where its level populations are measured (Figure 1). The study thus combines theoretical population-based discrimination with experimental characterization of the circuits proposed for its implementation.

## 2. Concept and Theory of the Qudit-Based Detector

### 2.1. Detection Concept

The proposed detector consists of a signal resonator capacitively coupled to a flux-tunable transmon operated as a multilevel qudit (Figure 1). The microwave field is stored in the resonator, and the transmon acts as the sensing element. The detection target is to distinguish specified input states through the excitation populations they produce in the qudit.

The qudit is initialized in its ground state |0〉 and interacts with the signal mode for a fixed time $t_{\mathrm{int}}$ at a selected external flux bias $\Phi_{\mathrm{ext}}$. This bias controls the detuning Δω between the qudit and the signal mode. The calculated detector response at the end of the interaction is the population vector

$$W\left(t_{\mathrm{int}},\Phi_{\mathrm{ext}}\right)=\left(W_{0},W_{1},W_{2},W_{3},W_{4},\ldots \right).$$

We compare Fock state and coherent resonator inputs with equal mean energy and use prescribed classical-field excitation as a separate reference. Their population dynamics depend on the input state, the qudit anharmonicity, the coupling and the selected detuning. At the illustrative operating points considered below, a Fock input produces a strong enhancement of a selected transmon population, while the coherent input and the classical drive give different population distributions. Discrimination is therefore based on comparing these distributions at a common interaction time. In the linear regime, the classical target-population bound provides a separate criterion for nonclassicality.

The proposed readout stage follows the interaction: the qudit is flux-tuned to a dispersive point, and its populations are estimated from repeated preparations and measurements. The calculations presented here describe the interaction-stage populations; their preservation during the flux ramp and their extraction through dispersive readout require calibration of the implemented protocol.

Within the Kerr model, the population response is governed by the anharmonicity γ, the coupling *g* and the detuning Δω, together with the input state and interaction time. The next subsection introduces the cosine transmon Hamiltonian used for spectroscopic fitting and its Kerr approximation used for the population dynamics. Section 2.3 then formulates the quantized-mode and classical-field descriptions of the signal–qudit interaction.

### 2.2. From the Transmon Circuit to the Kerr Qudit Model

The transmon enters the detector twice: as a circuit whose spectrum is measured and fitted, and as a dynamical multilevel system whose populations are the detector output. The full cosine Hamiltonian serves the first role, and its weakly anharmonic (Kerr) reduction serves the second. The point of this subsection is that the two are the same circuit, so that the parameters returned by the spectroscopic fit of Section 4 enter the population maps of Section 3 without any adjustable constant.

A flux-tunable transmon is described by the standard Hamiltonian

(1) $${\hat{H}}_{\mathrm{tr}}=4E_{C}{\left({\hat{n}}_{CP}-n_{g}\right)}^{2}-E_{J}\left(\Phi_{\mathrm{ext}}\right)\cos\hat{\varphi },\;\left[\hat{\varphi },{\hat{n}}_{CP}\right]=i,$$

where $E_{C}=e^{2}/\left(2C_{\Sigma }\right)$ is the charging energy and

$$E_{J}\left(\Phi_{\mathrm{ext}}\right)=E_{J\Sigma }\sqrt{\cos^{2}\left(\pi \Phi_{\mathrm{ext}}/\Phi_{0}\right)+d^{2}\sin^{2}\left(\pi \Phi_{\mathrm{ext}}/\Phi_{0}\right)}$$

is the flux-dependent effective Josephson energy of the SQUID, with $E_{J\Sigma }=E_{J1}+E_{J2}$ and junction asymmetry *d*.

For the spectroscopic analysis of Section 4, Equation (1) is diagonalized numerically in the charge basis without expanding the Josephson potential. In the transmon regime, $E_{J}/E_{C}\gg 1$, the same circuit reduces to a weakly anharmonic oscillator. Keeping the leading nonlinear contribution gives

(2) $${\hat{H}}_{\mathrm{tr}}≃\hbar \omega_{p}{\hat{b}}^{†}\hat{b}-\frac{E_{C}}{12}{\left(\hat{b}+{\hat{b}}^{†}\right)}^{4},\;\omega_{p}=\frac{\sqrt{8E_{J}\left(\Phi_{\mathrm{ext}}\right)E_{C}}}{\hbar }.$$

Equation (2) is the working model of the detector. Its anharmonicity is fixed by the charging energy, $\gamma =-E_{C}/\left(2\hbar \right)$ in the notation of Section 2.3; its transition frequency is set by the flux through $\omega_{p}\left(\Phi_{\mathrm{ext}}\right)$; and the coupling *g* to the signal mode is introduced below. These are precisely the quantities $E_{C}$, $E_{J\Sigma }$, *d*, and *g* that the fit of the two-tone spectra returns (Table A1 and Table A2). The explicit reduction from the full cosine Hamiltonian, together with the charge-basis representation used for numerical fitting, is given in Appendix A.

**Figure A1:**
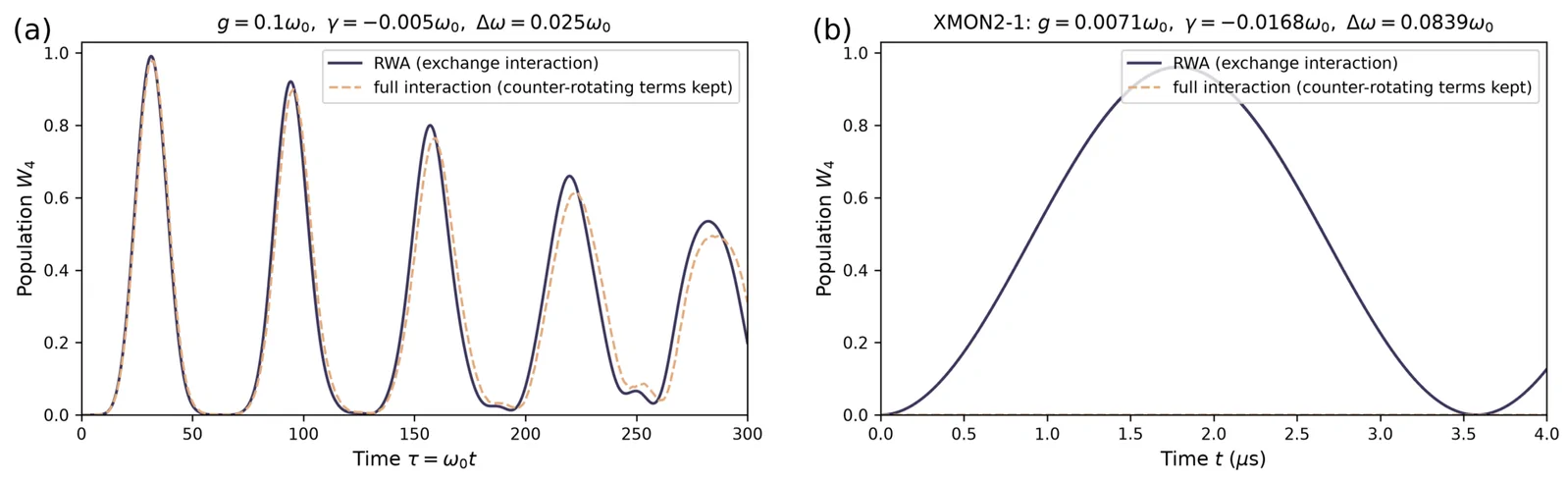
Population of the target level n=4 for the Fock-state input $k_{0}=4$ computed with the RWA Hamiltonian of Equation (4) (solid) and with the full interaction of Equation (A4) (dashed): (**a**) strong-coupling operating point of Figure 4, Figure 5, Figure 6 and Figure 7; (**b**) measured parameters of device XMON2-1 (Figure 8), averaged over 20 ns.

The transmon is capacitively coupled to a resonator mode through its charge operator. Depending on the phase convention chosen for the resonator quadrature, the interaction can be written equivalently as

(3) $${\hat{H}}_{\mathrm{int}}\propto {\hat{n}}_{CP}\left(\hat{a}+{\hat{a}}^{†}\right)\;\mathrm{or}\;{\hat{H}}_{\mathrm{int}}\propto i{\hat{n}}_{CP}\left({\hat{a}}^{†}-\hat{a}\right),$$

where $\hat{a}$ and ${\hat{a}}^{†}$ are the resonator ladder operators [29,37,42]. The two forms are related by a phase rotation of the resonator mode and lead to the same observable spectrum. In the circuit-QED regime $g\ll \omega_{0},\omega_{01}$, the rotating-wave approximation retains the exchange interaction between resonator and transmon excitations. This gives the multilevel signal–qudit Hamiltonian introduced in the next section and used throughout the population-dynamics analysis. The accuracy of the rotating-wave step is verified in Appendix A by propagating the full interaction, with the counter-rotating terms retained, for both operating points used in this paper (Figure A1): at the strong-coupling point $g=0.1\omega_{0}$, the height and the position of the first population maximum change by 1% and 2%, respectively, and at the measured device parameters, the two calculations coincide within $5\times 10^{-3}$.

### 2.3. Qudit Interacting with a Quantized or a Classical Signal Field

We now formulate the two dynamical models that are compared throughout this work. In the fully quantized description, both the signal mode and the qudit are quantum subsystems, and the Hamiltonian has the following form [38]:

(4) $$\hat{H}=\hbar \omega_{0}\left({\hat{n}}_{a}+\frac{1}{2}\right)+\hbar \left(\omega_{0}+\Delta \omega \right)\left({\hat{n}}_{b}+\frac{1}{2}\right)+\hbar \gamma {\hat{n}}_{b}\left({\hat{n}}_{b}+1\right)+\hbar \frac{g}{2}\left({\hat{b}}^{†}\hat{a}+\hat{b}{\hat{a}}^{†}\right),$$

where the operators ${\hat{n}}_{a}$ and ${\hat{n}}_{b}$ stand for the number of photons in the field mode and the number of excitations in the qudit, respectively, $\hat{a}$ and $\hat{b}$ are the annihilation operators of the same subsystems, *g* is the interaction strength, and γ characterizes the nonlinearity of the qudit. This form is obtained by applying the RWA to Equations (2) and (3), with $\omega_{p}=\omega_{0}+\Delta \omega$ and $\gamma =-E_{C}/\left(2\hbar \right)$. The dynamics is solved exactly, and the observables of interest—the qudit level populations $W_{n}\left(t\right)$—are then calculated by tracing over the field mode.

In the coarse classical description, the same transmon-based qudit is driven by a prescribed c-number field, and the Hamiltonian reads

(5) $$\hat{H}=\hbar \left(\omega_{0}+\Delta \omega \right){\hat{n}}_{b}+\hbar \gamma {\hat{n}}_{b}\left({\hat{n}}_{b}+1\right)+\frac{\hbar G}{\sqrt{2}}\cos\omega_{0}t\left({\hat{b}}^{†}+\hat{b}\right),$$

where *G* is the strength of the interaction with the classical field. Solving the nonstationary Schrödinger equation leads to the following system of equations for the amplitudes of the transmon states populated by the classical drive:

(6) $$i\hbar {\dot{C}}_{n}\left(t\right)=\hbar n\left(\gamma (n+1)+\Delta \omega \right)C_{n}\left(t\right)-\frac{\hbar G}{2}\left(\sqrt{\frac{n+1}{2}}\;C_{n+1}\left(t\right)+\sqrt{\frac{n}{2}}\;C_{n-1}\left(t\right)\right).$$

The Hamiltonians of Equations (4) and (5) describe the detector-stage interaction between the calibrated transmon qudit and a signal mode. In both the quantized and the classical cases, the experimentally relevant observables are the qudit populations $W_{n}\left(t\right)$. The quantized and classical excitations are compared at equal initial field energy, so that the following relation between the classical and quantum interaction strengths holds:

(7) $$G=g\sqrt{2k_{0}},$$

with $k_{0}$ being the mean number of photons in the quantized field mode at the initial time (for a Fock state, it is the photon number). In such a case, the difference between the classical and quantum impact is encoded in the population dynamics. For convenience, dimensionless parameters normalized to the resonator frequency $\omega_{0}$ are used below, and time is measured in units of $1/\omega_{0}$.

The three inputs compared in this work form a hierarchy within one and the same model. Replacing the field operator in Equation (4) by its mean value in a coherent state, $\hat{a}\to \sqrt{k_{0}}\;e^{-i\omega_{0}t}$, turns the exchange term into the classical drive of Equation (5) with exactly the amplitude $G=g\sqrt{2k_{0}}$ of Equation (7). The classical drive is therefore the mean-field limit of the quantized coherent input: it has the same energy at t=0 and the same coupling, but its amplitude is undepleted when excitations are transferred to the qudit, whereas a quantized field loses one photon per transferred excitation. The Fock state, in turn, differs from the coherent state only by its photon-number statistics. The population dynamics thus isolates two effects, depletion and photon-number statistics, with everything else held fixed.

## 3. Population Dynamics of Classical and Quantized Microwave Fields

Let us first highlight the fundamental feature of the qudit excitation induced by classical and quantized fields. In the explicitly resonant case, the classical drive gradually excites the transmon, with higher and higher states becoming significantly populated. The distribution over transmon states can be shown to be Poissonian, with a mean excitation number that grows quadratically with time. In contrast, the interaction with a quantized field is limited by the initial number of photons in the field mode, so that the number of qudit levels involved in the excitation is bounded. Moreover, it turns out that for a definite number of photons in the quantized field, the distribution of qudit excitations follow a binomial law. Such a case describes the full transfer of the field photons to the excitation of the transmon level with the corresponding number. This periodic complete transfer, with the divergence of the qudit excitation number vanishing at the transfer times, has no classical counterpart and is the first signature exploited below.

### 3.1. Non-Resonant Linear Regime: Poissonian Versus Binomial Fingerprints

Exact resonance, however, is rarely achievable in practice. For the off-resonant case with Δω≠0 but without nonlinearity in the system (γ=0), we have found that the distribution over the transmon states populated by the classical drive remains Poissonian:

(8) $$W_{n}=\frac{1}{n!}{\left(\bar{n}\right)}^{n}e^{-\bar{n}},$$

but the mean number of excitations $\langle n(t)\rangle$ is now bounded and oscillates in time:

(9) $$\langle n(t)\rangle =\frac{G^{2}}{2\Delta \omega^{2}}\sin^{2}\frac{\Delta \omega t}{2}.$$

For the quantized field, we obtain the following time dependence of the mean number of excitations $\langle n(t)\rangle$:

(10) $$\langle n(t)\rangle =\langle {\hat{a}}^{†}\hat{a}\rangle \frac{g^{2}}{g^{2}+\Delta \omega^{2}}\sin^{2}\frac{\sqrt{g^{2}+\Delta \omega^{2}}\;t}{2},$$

where $\langle {\hat{a}}^{†}\hat{a}\rangle$ denotes the initial mean number of photons in the quantized field mode.

**Figure 2:**
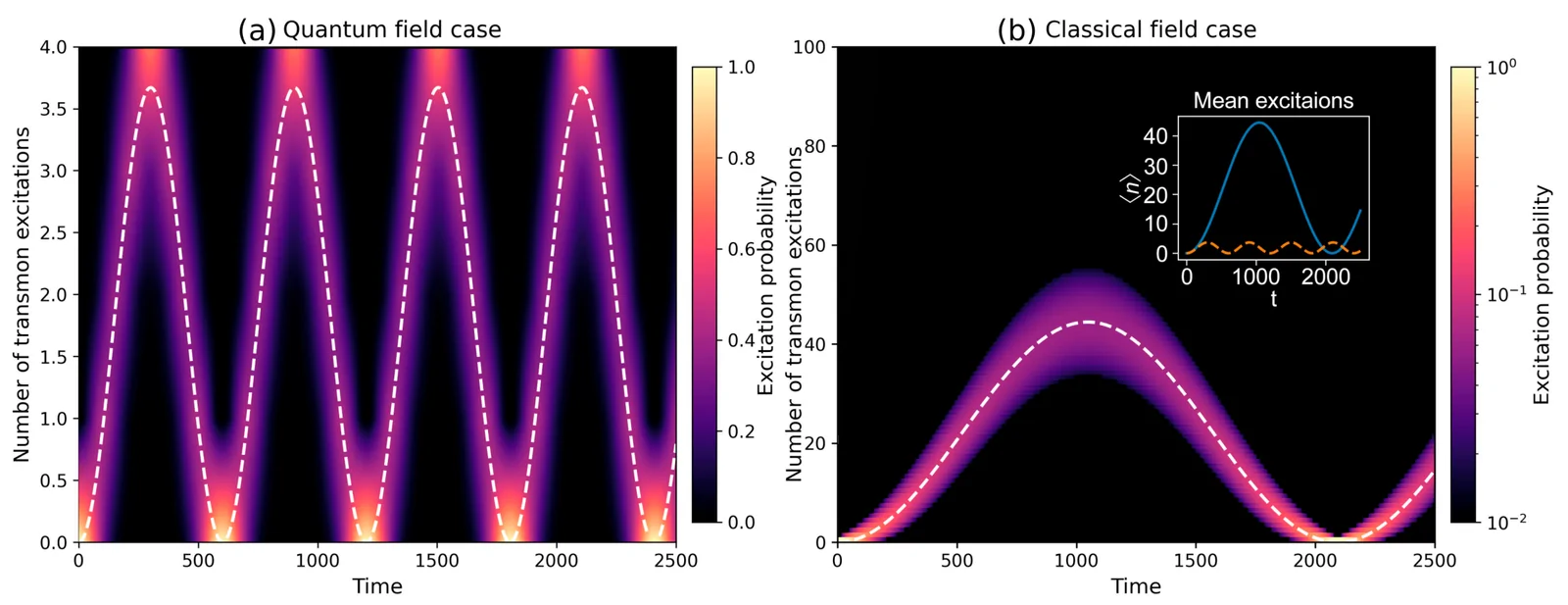
Distribution (color-coded) of the probability of transmon state excitation versus time in the linear regime (γ=0): (**a**) quantized-field case with $g=0.01\omega_{0}$, $\Delta \omega =0.003\omega_{0}$ for the initial Fock state $k_{0}=4$; (**b**) classical-drive case with $G=0.028\omega_{0}$, $\Delta \omega =0.003\omega_{0}$. White dashed lines show the time dependence of the mean number of transmon excitations. The inserted image shows the difference in the dynamics of the mean number of transmon excitations in the case of quantum (orange dashed line) and classical fields (blue line). Time is presented in arbitrary units $\tau =\omega_{0}t_{\mathrm{phys}}$.

Let us analyze this behavior in detail for an interaction strength close to the experimentally obtained value and for different detunings, which can be varied by applying a magnetic flux to the qudit. If the detuning is much larger than the interaction strength *g*, the qudit excitation dynamics observed in the classical and quantized cases are found to be almost the same, so a detection protocol requires a different regime. Indeed, a very important and strong difference in the excitation dynamics induced by classical and quantized fields is found when the detuning is small in comparison to the interaction strength *g*. The regime of small detuning is presented in Figure 2. In the classical case, the small detuning leads to a very small oscillation frequency of the mean excitation number, while the oscillation amplitude, inversely proportional to the detuning squared, may become very large. As a result, the oscillations are very slow and the qudit remains strongly excited during almost all of the evolution (Figure 2b). For the quantized field, Rabi-like oscillations of the mean excitation number take place, and neither the frequency nor the amplitude changes significantly at small detunings. The highest possible excitation corresponds to the level with $n=k_{0}$, which is periodically strongly populated (Figure 2a). Since the timescale is presented in periods of the resonator frequency, a lot of oscillations can be observed within the characteristic dephasing time $T_{2}^{*}$.

A deeper insight comes from the analysis of the time-dependent distribution over the excited transmon states, which in the case of the quantized field is found to remain binomial (as in the resonant case), but with different time dependencies of the parameters *p* and *q*. For a field with an initial photon number $k_{0}$, the probability of observing *n* transmon excitations is characterized by the following binomial distribution:

(11) $$W_{n,k_{0}-n}=C_{k_{0}}^{n}p^{n}q^{k_{0}-n},$$

where

(12) $$p=\frac{g^{2}}{g^{2}+\Delta \omega^{2}}\sin^{2}\frac{\sqrt{g^{2}+\Delta \omega^{2}}\;t}{2},$$

(13) $$q=1-p=\cos^{2}\frac{\sqrt{g^{2}+\Delta \omega^{2}}\;t}{2}+\frac{\Delta \omega^{2}}{g^{2}+\Delta \omega^{2}}\sin^{2}\frac{\sqrt{g^{2}+\Delta \omega^{2}}\;t}{2},$$

and

(14) $$C_{k_{0}}^{n}=\frac{k_{0}!}{n!(k_{0}-n)!}$$

is the binomial coefficient.

The binomial law has a direct consequence for the classical benchmark. For γ=0, the qudit and the signal mode form a pair of linearly coupled oscillators, and such a coupling maps a coherent state |α〉 of the field onto a coherent state of the qudit with mean excitation number $\bar{n}\left(t\right)={\left|\alpha \right|}^{2}p\left(t\right)$, with p(t) given by Equation (12); the qudit populations are then Poissonian, $W_{n}=e^{-\bar{n}}{\bar{n}}^{\;n}/n!$, which is the quantized counterpart of Equations (8) and (9). Any field with a positive Glauber–Sudarshan *P*-function is a mixture of coherent states, so its population vector is a mixture of Poissonians, and the population of any level is bounded by the maximum of the Poisson distribution over its mean:

(15) $$W_{n}^{\mathrm{cl}}\left(t\right)\leq \max_{\bar{n}}\frac{e^{-\bar{n}}{\bar{n}}^{\;n}}{n!}=\frac{e^{-n}n^{n}}{n!},\;W_{2}^{\mathrm{cl}}\leq 0.271,\;W_{3}^{\mathrm{cl}}\leq 0.224,\;W_{4}^{\mathrm{cl}}\leq 0.195.$$

**Figure 3:**
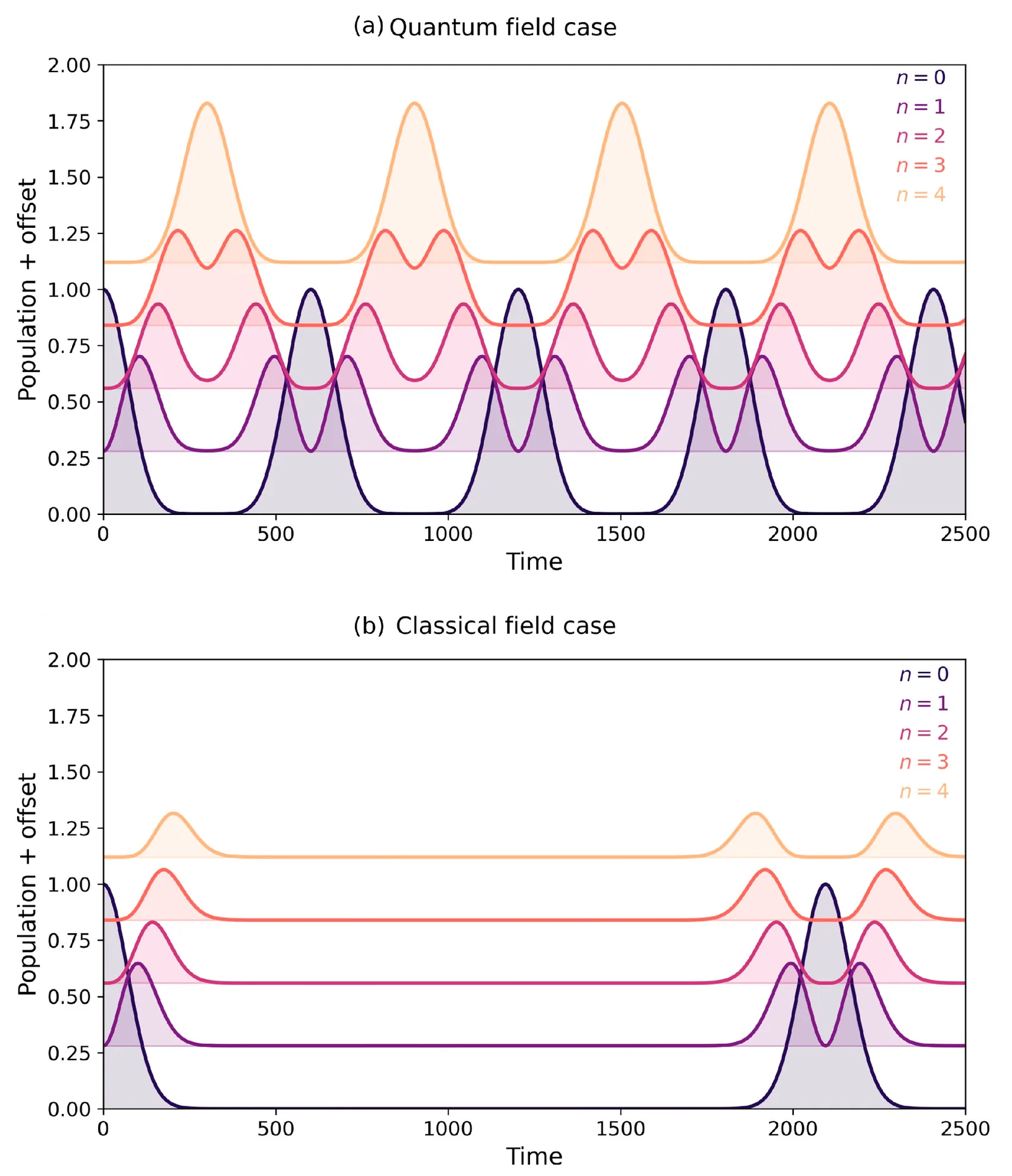
Dynamics of the populations of the qudit levels with n=0,1,2,3,4 in the linear regime (γ=0): (**a**) quantized-field case with $g=0.01\omega_{0}$, $\Delta \omega =0.003\omega_{0}$, for the initial Fock state $k_{0}=4$; (**b**) classical-drive case with the same initial energy, $G=0.028\omega_{0}$, $\Delta \omega =0.003\omega_{0}$. An offset was added for clarity.

The bound holds for every classical field, for every detuning and interaction time in the linear regime, and it is reached by the c-number drive of Equation (5) at the instants when $\bar{n}\left(t\right)=n$. The Fock state, by contrast, gives $W_{k_{0}}=p^{k_{0}}$, which approaches unity at resonance and equals 0.71 for the detuning of Figure 3. In this regime, a target-level population above the bound of Equation (15) therefore witnesses a negative *P*-function, i.e., the nonclassicality of the signal field, and requires neither knowledge of the field energy nor a specific classical reference.

During the interaction with a four-photon quantized field, a well-pronounced population of the highest possible qudit state with n=4 is periodically repeated in time. The probability of exciting the same fourth level by the classical drive appears to be quite low due to the features of the Poissonian distribution (see Figure 3). In the classical case, the population is transferred to much higher levels, so that the revival time is found to be much larger than in the quantized-field case (see, for comparison, the inset in Figure 2b). Thus, to detect the nonclassicality of the field in this regime, we should focus on the population of the fourth qudit state. The high value of this population and its further growth with time up to unity witness the nonclassical nature of the affecting field (Equation (15)). The specific feature of the quantized-field impact consists in the predominant population of the lowest (initial) and the highest possible qudit levels, periodically replacing each other in time. Such population dynamics is a signature of the quantum nature of the field even when the initial energy or the mean photon number of the field mode is unknown in advance.

### 3.2. Nonlinearity and Optimal Detuning: Selective Enhancement of a Target Level

Let us now consider the realistic case, in which the nonlinearity of the transmon should be taken into account. If the coupling between the transmon and the electromagnetic field is weak in comparison with the nonlinear interaction, the nonlinear effects dominate and mainly determine the dynamics of the system for either a quantized or a classical field.

A significant difference between the interaction with quantized and classical fields appears in the strong-coupling regime. In this regime, the populations of the qudit states oscillate in time for both types of fields; however, for the classical drive, the oscillation frequency is determined by the detuning and the nonlinearity parameter. For the quantized field, it strongly depends on the coupling strength *g*. Thus, a strong difference in the excitation dynamics is expected.

Contrast between the excitation scenarios is seen when the detuning is specifically chosen to compensate the nonlinear effects for a certain qudit level and to enhance its excitation. The value of this optimal detuning tuned to a level number *n* is found to be the following:

(16) $$\Delta \omega_{\mathrm{opt}}^{\left(n\right)}=-\gamma \left(n+1\right).$$

**Figure 4:**
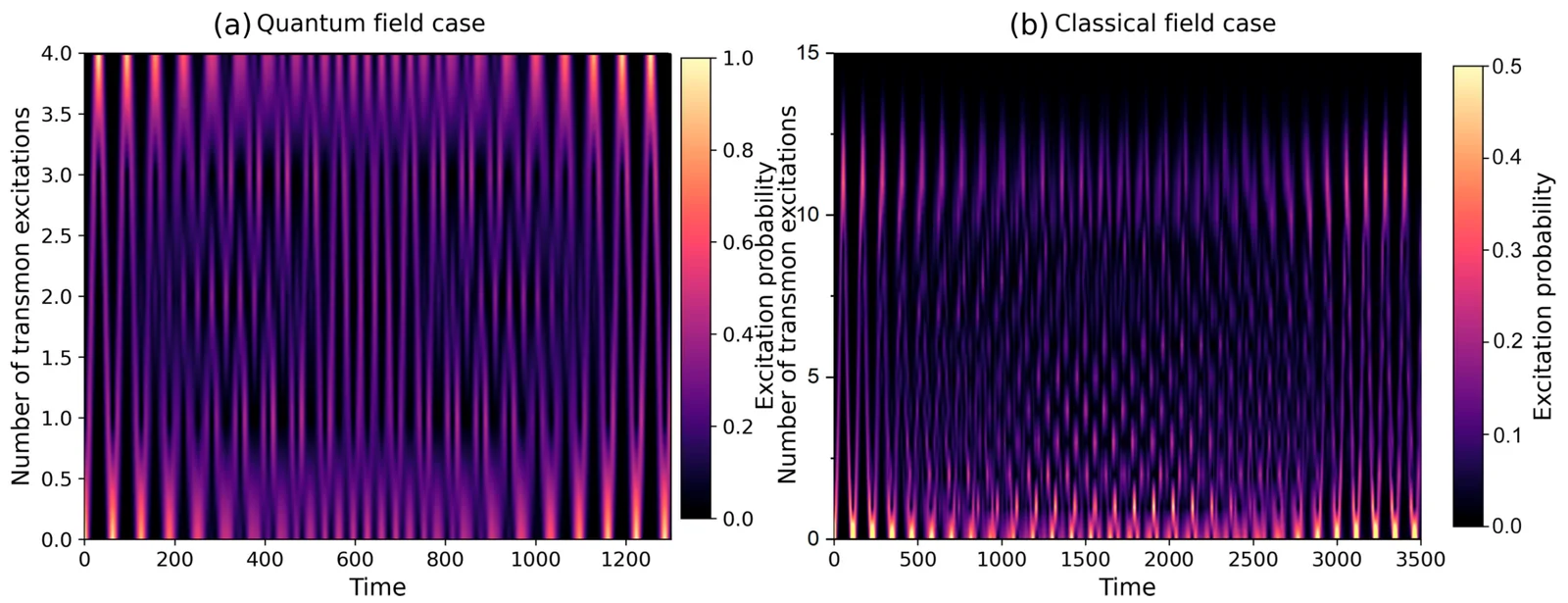
Distribution (color-coded) of the probability of transmon state excitation versus time in the nonlinear regime: (**a**) quantized-field case with $g=0.1\omega_{0}$, $\gamma =-0.005\omega_{0}$, $\Delta \omega =0.025\omega_{0}$ for the initial Fock state $k_{0}=4$; (**b**) classical-drive case with $G=0.283\omega_{0}$, $\gamma =-0.005\omega_{0}$, $\Delta \omega =0.025\omega_{0}$.

**Figure 5:**
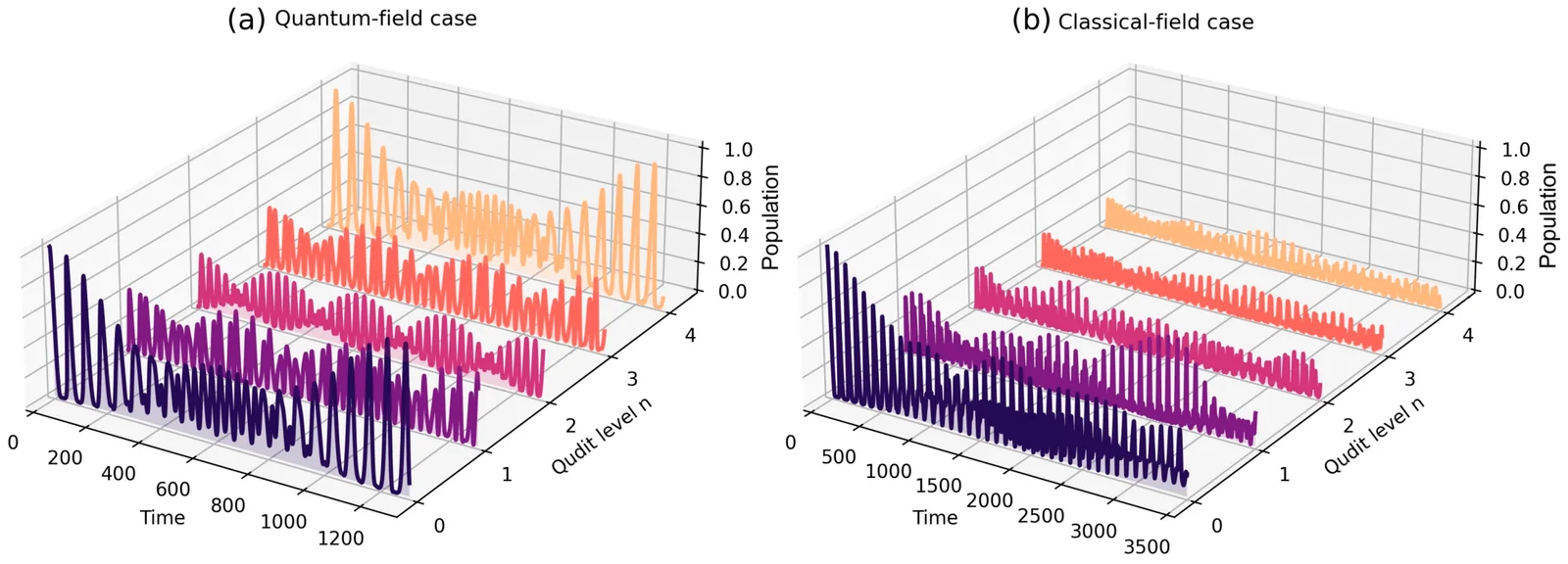
Dynamics of the populations of the qudit levels with n=0,1,2,3,4 in the nonlinear regime: (**a**) quantized-field case with $g=0.1\omega_{0}$, $\gamma =-0.005\omega_{0}$, $\Delta \omega =0.025\omega_{0}$, for the initial Fock state $k_{0}=4$; (**b**) classical-drive case with the same initial energy, $G=0.283\omega_{0}$, $\gamma =-0.005\omega_{0}$, $\Delta \omega =0.025\omega_{0}$. The selective enhancement of $W_{4}\left(t\right)$ in panel (**a**) is the most relevant detector signature.

By choosing a value of the optimal detuning proper for the highest level that can be populated by the quantized field (the level with n=4 in our case), it is possible to provide its strong excitation, periodically repeating in time (see Figure 4). The classical drive spreads population over a much larger number of qudit states, with only the ground state retaining a high probability. A quantitative analysis shows that already during the first oscillation, the target state is populated with almost unit probability in the quantized case (see Figure 5a). This rapid transfer occurs at the illustrative strong-coupling point. The weaker-coupling case and its sensitivity to dissipation are examined in Section 3.4 and Appendix E. In the case of the classical drive, the population of the fourth state—and of all states except the ground and, partly, the first one—is strongly suppressed, and the characteristic oscillation time is several times larger. As a result, the excitation dynamics in quantized and classical fields differs dramatically. The observed transfer of all the energy from the quantized field to the qudit excitation, together with the resulting distribution of the qudit level populations, allows one to extract information about the initial quantum state of the field. The established link between the field and qudit characteristics—photon numbers and excitation probabilities $\left\{W_{n}\right\}$—is what enables the detector design of Section 2.1.

### 3.3. Coherent States with a Small Mean Photon Number

The comparison above contrasts the two limiting descriptions of the signal field: the coarse classical (c-number) drive and a strongly nonclassical Fock state. A practically important intermediate case is a *quantized coherent state* with a small mean photon number,

(17) $$|\alpha \rangle =e^{-{\left|\alpha \right|}^{2}/2}\sum_{k}\frac{\alpha^{k}}{\sqrt{k!}}|k\rangle ,\;\bar{k}={\left|\alpha \right|}^{2},$$

which is the closest quantum counterpart of a classical monochromatic field and is exactly the state produced by an attenuated microwave generator. A meaningful detector of few-photon nonclassical fields must therefore distinguish a Fock state both from the classical drive and from a coherent state of the same mean energy, $\bar{k}=k_{0}$. Since the coherent state is a Poissonian superposition of Fock components, its evolution under the Hamiltonian of Equation (4) is obtained by propagating each excitation-number sector independently, and the qudit populations follow by tracing over the field mode.

**Figure 6:**
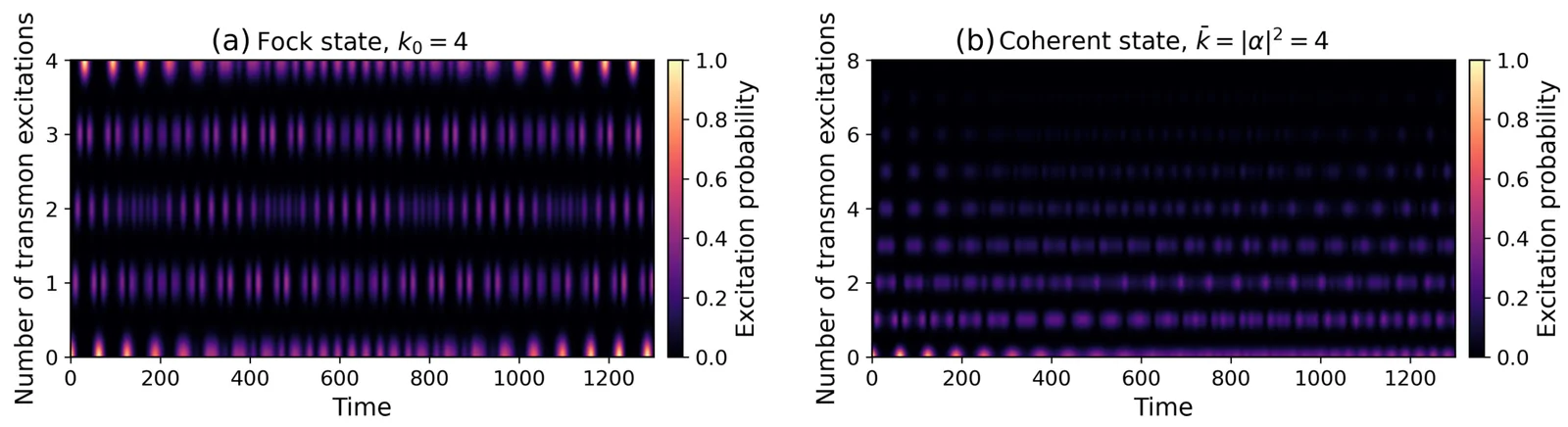
Distribution (color-coded) of the probability of transmon state excitation versus time in the nonlinear regime ($g=0.1\omega_{0}$, $\gamma =-0.005\omega_{0}$, $\Delta \omega =0.025\omega_{0}$) for two quantized input states of the same mean energy: (**a**) Fock state with $k_{0}=4$; (**b**) coherent state with $\bar{k}={\left|\alpha \right|}^{2}=4$. For the Fock state, the excitation periodically concentrates in the target level n=4 and levels with n>4 remain empty; for the coherent state, the excitation spreads over the qudit ladder (including n>4), and every individual level keeps a population well below unity.

Figure 6 compares the qudit excitation dynamics induced by a Fock state with $k_{0}=4$ and by a coherent state with $\bar{k}={\left|\alpha \right|}^{2}=4$ at the same operating point as in Figure 4 and Figure 5, i.e., at the optimal detuning $\Delta \omega_{\mathrm{opt}}^{\left(4\right)}=0.025\omega_{0}$ for the target level n=4. The contrast is striking. For the Fock state, the population is periodically and almost completely concentrated in the target level n=4; the levels with n>4 remain strictly empty because the field initially contains exactly four photons. For the coherent state, the same operating point produces a qualitatively different pattern: the excitation is distributed over many qudit levels, including the levels n>4 populated by the higher Fock components of the coherent superposition, and the population of every individual level stays well below unity.

**Figure 7:**
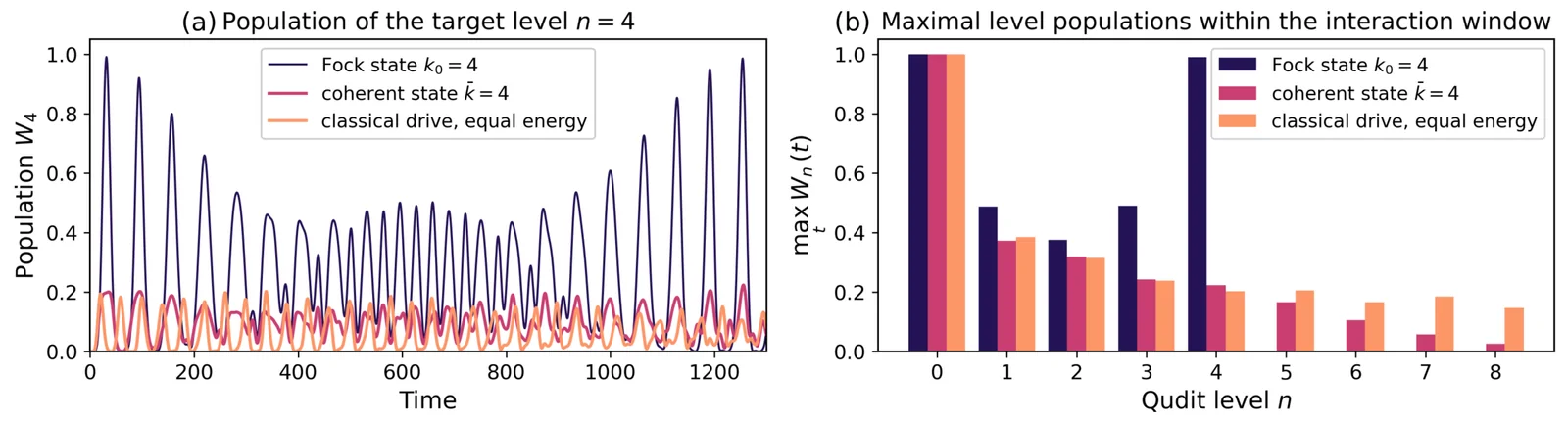
Population-based discrimination of input fields of equal mean energy in the nonlinear regime ($g=0.1\omega_{0}$, $\gamma =-0.005\omega_{0}$, $\Delta \omega =0.025\omega_{0}$): (**a**) population $W_{4}\left(t\right)$ of the target level n=4 for the Fock state $k_{0}=4$, the coherent state $\bar{k}=4$, and the classical drive of equal energy, $G=g\sqrt{2k_{0}}\approx 0.283\omega_{0}$; (**b**) maximal level populations $\max_{t}W_{n}\left(t\right)$ reached within the interaction window $\omega_{0}t\in \left[0,1300\right]$ for the same three inputs at different points in time (so $\sum_{n}\max_{t}W_{n}\left(t\right)>1$). The Fock state yields a near-unit population of the target level and no excitation beyond it; the coherent state stays close to the Poissonian weight of its resonant four-photon component (Equation (18)); the classical drive spreads the excitation to much higher levels. The population vectors of the three inputs at the common time $\tau^{*}=31.4$ are listed in Table 1.

The discrimination becomes quantitative when one focuses on the population of the target level. Figure 7a shows $W_{4}\left(t\right)$ for the three types of input of equal mean energy: the Fock state $k_{0}=4$, the coherent state $\bar{k}=4$, and the classical drive with $G=g\sqrt{2k_{0}}$. For the Fock state, $W_{4}$ periodically reaches values close to unity ($\max_{t}W_{4}\approx 0.99$ in the simulated window). For the coherent state, the target level can be reached only by the Fock components with k≥4 photons, so that $W_{4}$ is bounded by the Poissonian weight of these components,

(18) $$W_{4}\leq P_{\bar{k}}\left(k\geq 4\right)=1-e^{-\bar{k}}\sum_{k=0}^{3}\frac{{\bar{k}}^{k}}{k!}\approx 0.57\;\left(\bar{k}=4\right).$$

The components with different *k* are transferred with different exchange rates and reach their maxima at different times, so the actual maximum stays far below this bound: at the operating point of Figure 7, $\max_{t}W_{4}\approx 0.22$, close to the weight $P_{\bar{k}}\left(4\right)=e^{-\bar{k}}{\bar{k}}^{4}/4!\approx 0.195$ of the k=4 component alone, which dominates the transfer at the detuning tuned to the four-photon sector; the small excess above 0.195 is the contribution of the k≥5 components. The classical drive produces a comparable $\max_{t}W_{4}\approx 0.2$, but with a very different distribution over the remaining levels.

Figure 7b summarizes the resulting population fingerprints through the maximal populations $\max_{t}W_{n}\left(t\right)$ reached within the interaction window. The three classes of input fields of equal energy are clearly distinguishable: (i) the Fock state produces a near-unit selective enhancement of the target level $n=k_{0}$ and strictly zero population beyond it; (ii) the coherent state excites the target level with a probability given by the Poisson-weighted sum of binomial contributions from all components with k≥4 photons in the initial coherent field state; the Fock components with k<4 leave the target level unexcited, so its maximal population stays far below the weight $P_{\bar{k}}\left(k\geq 4\right)$ of the contributing components. Owing to this binomial character of the transmon population, the excitation pattern at each instant of time is strongly non-monotonous over *n*, with some transmon levels remaining almost unpopulated; (iii) the classical drive produces a broad, slowly decreasing population profile extending to levels far above $n=k_{0}$. Thus, population-based readout distinguishes a few-photon Fock state both from the coarse classical drive and from the “most classical” quantized state of the same energy, while the tail of the population distribution at $n>k_{0}$ additionally separates the coherent state from the classical drive. We stress that this three-way discrimination is achieved at a single operating point, without sweeping the detuning, and therefore fits directly into the two-stage protocol of Section 2.1.

The maxima in Figure 7b are reached at different times and display the reach of each input over the ladder. The detector, however, reads the population vector at one interaction time. Table 1 lists the three vectors at the single time $\tau^{*}=31.4$ at which the Fock-state transfer to n=4 is complete. The three inputs remain clearly distinct: the total-variation distances $D_{TV}=\frac{1}{2}\sum_{n}\left|W_{n}-W_{n}^{\prime }\right|$ are 0.79 (Fock–coherent), 0.98 (Fock–classical), and 0.76 (coherent–classical), and the classical drive is recognized at once by the 65% of its population that has left the first nine levels.

The total-variation distance quantifies the distinguishability of two input fields through their normalized qudit population distributions at a common interaction time. Physically, it is the largest difference between the probabilities of obtaining an outcome within any chosen subset of qudit levels. Thus, $D_{\mathrm{TV}}=0$ means identical population distributions, whereas $D_{\mathrm{TV}}=1$ means non-overlapping readout outcomes. For two equally likely input hypotheses, an ideal single-shot population measurement followed by the optimal decision rule gives the minimum error probability $P_{\mathrm{err}}^{\left(\mathrm{pop}\right)}=\frac{1-D_{\mathrm{TV}}}{2}$. The sum must include all readout outcomes, with unresolved higher levels retained as an additional outcome.

### 3.4. Operating Point at the Measured Device Parameters

**Figure 8:**
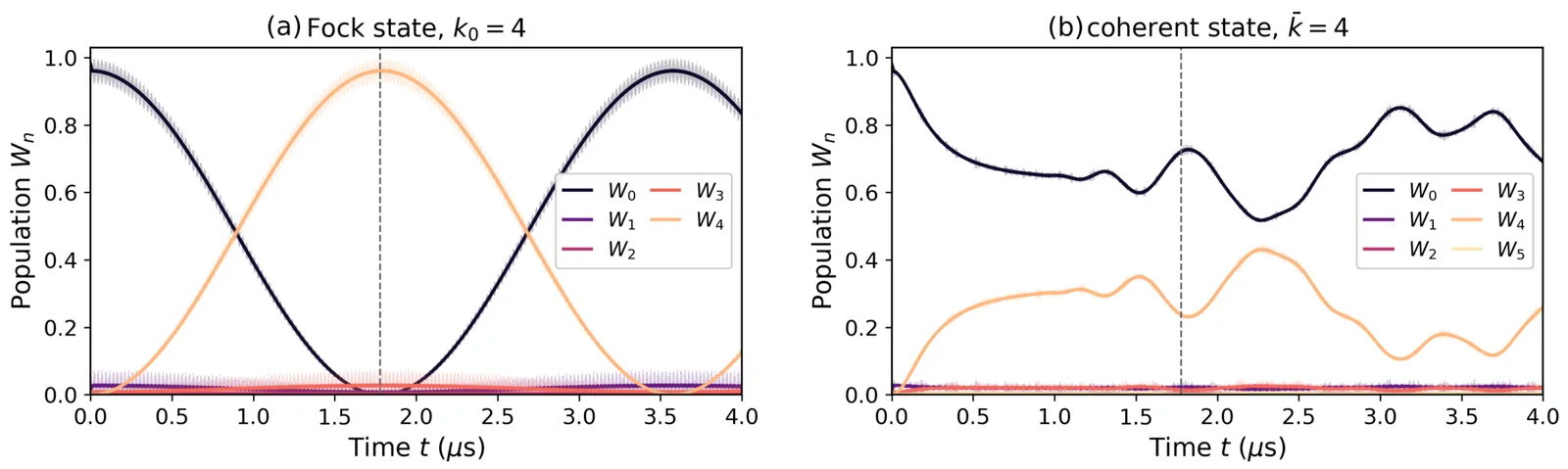
Population dynamics at the measured parameters of device XMON2-1 ($\omega_{0}/2\pi =6.494$ GHz, $g=0.0071\omega_{0}$, $\gamma =-0.0168\omega_{0}$) at the detuning tuned to the four-photon sector, $\Delta \omega =\Delta \omega_{\mathrm{opt}}^{\left(4\right)}=0.0839\omega_{0}$ (545 MHz): (**a**) Fock state $k_{0}=4$; (**b**) coherent state $\bar{k}=4$; Dashed line: $t^{*}=1.8$ μs, the first maximum of the Fock-state transfer.

The operating point of Figure 4, Figure 5, Figure 6 and Figure 7 was chosen to display the amplitude contrast in its purest form and corresponds to g/|γ|=20. Our measured devices (Section 4) lie in the opposite regime. For device XMON2-1, g/2π=46 MHz and $E_{C}/h=218$ MHz, i.e., $g=0.0071\omega_{0}$ and $\gamma =-E_{C}/\left(2\hbar \right)=-0.0168\omega_{0}$ in the units of Equation (4), so that g/|γ|=0.42. The detuning tuned to the four-photon sector, Equation (16), is $\Delta \omega_{\mathrm{opt}}^{\left(4\right)}=5\left|\gamma \right|=0.0839\omega_{0}$, i.e., $\Delta \omega_{\mathrm{opt}}^{\left(4\right)}/2\pi =545$ MHz. Figure 8 presents the population dynamics for two quantized inputs of equal mean energy: a Fock state with $k_{0}=4$ and a coherent state with $\bar{k}=4$. Both cases are calculated using the Hamiltonian of Equation (4), with physical time shown for $\omega_{0}/\left(2\pi \right)=6.494\;\mathrm{GHz}$.

For the Fock input, the numerical solution of Equation (4) shows that the population is exchanged between the ground state and the target level n=4, while the intermediate levels remain weakly populated (Figure 8a). For the coherent input in Figure 8b, the target-level population remains substantially below unity, with $\max_{t}W_{4}\left(t\right)\approx 0.43$ within the simulated interval. The dashed lines in Figure 8a,b indicate the common interaction time $t^{*}=1.8\;\mu s$, corresponding to the first maximum of the Fock-state transfer. At this time, $W_{4}\left(t^{*}\right)\approx 0.96$ for the Fock input and $W_{4}\left(t^{*}\right)\approx 0.24$ for the coherent input. The target-level population therefore distinguishes these two equal-energy inputs at a fixed interaction time. The populations in Figure 8 provide a unitary reference. Appendix E presents a phenomenological dissipative calculation in which, under the stated noise assumptions, the Fock–coherent target-population contrast at the same interaction time decreases from approximately 0.72 to 0.24.

## 4. Experimental Characterization and Parameter Calibration

To connect the operating points identified in Section 3 with experimentally realizable hardware, we characterize several transmon–resonator systems and determine the relevant coupling, detuning, and coherence scales.

### 4.1. Experimental Characterization of the Transmon Qudit

**Figure 9:**
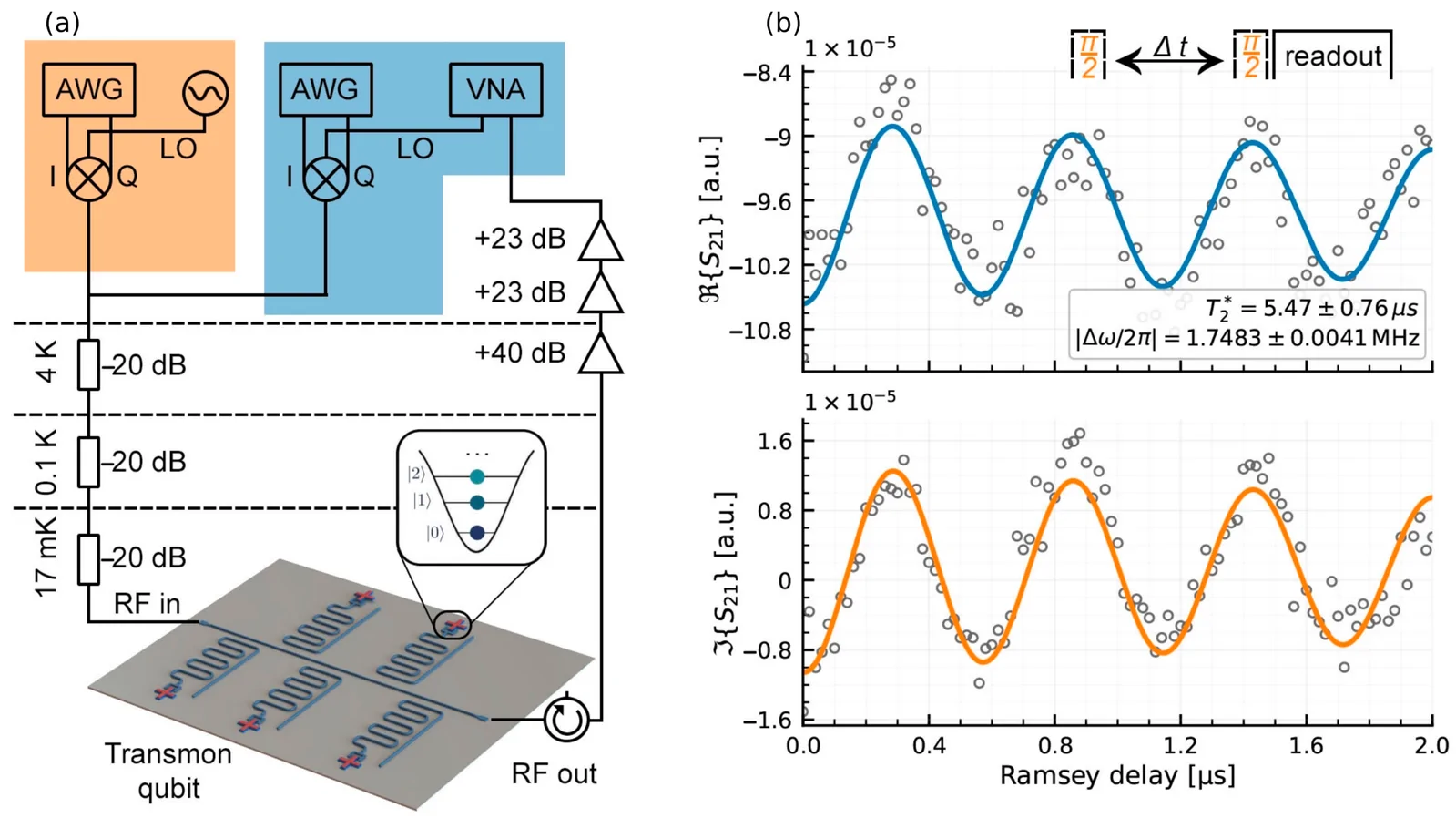
Experimental setup and methods: (**a**) schematic diagram of the transmon qudit sensor and readout resonator (coplanar waveguide) connected to the employed microwave setup designed to measure the complex transmission coefficient $S_{21}$ of the readout resonator; (**b**) example of experimentally demonstrated Ramsey oscillations on the fundamental transition |0〉→|1〉 for the real and imaginary parts of $S_{21}$ as an initial calibration of the qudit sensor (transmon–resonator system 1, see Table A2).

Our qudit is a common tunable-frequency superconducting transmon [43,44] (Josephson plasma frequencies $\omega_{p}/2\pi \approx 4.87$–7.44GHz at the sweet spot $\Phi_{\mathrm{ext}}=0$) operated deliberately beyond the two-level approximation, i.e., as a multilevel anharmonic oscillator (qudit) [36,37,45,46]. This architecture combines a relatively low anharmonicity—advantageous for accessing and resolving higher transitions—with a large electric dipole moment, which enhances sensitivity to local microwave fields in the coupled resonator. Figure 9 introduces the experimental platform and the basic time-domain protocol used in our work (see Appendix C for details). Time-domain control and readout are implemented using a standard pulse sequence: shaped microwave pulses address the desired transition(s), and the qudit state is measured via the coupled readout resonator with time-resolved acquisition (Figure 9a).

As an initial calibration of the qudit sensor, we perform Ramsey interferometry on the fundamental transition |0〉→|1〉 (Figure 9b). A pair of π/2 pulses separated by a variable Ramsey delay Δt produces oscillations in the measured population of the addressed states, with a decaying envelope that yields the inhomogeneous dephasing time $T_{2}^{*}$. Fitting the data to a decaying cosine, we extract $T_{2}^{*}=\left(5.47\pm 0.76\right)\;\mu s$ and a residual detuning magnitude $|\Delta \omega_{d}|/2\pi =\left(1.748\pm 0.004\right)\;\mathrm{MHz}$. These measurements characterize coherence on the fundamental transition. Their implications for the multilevel population contrast are examined in Appendix E, where a phenomenological calculation shows substantial contrast reduction under the stated noise assumptions.

### 4.2. Two-Tone Spectroscopy and Parameter Extraction

Two distinct microwave tones are used during the spectroscopic characterization. A fixed probe tone at frequency $\omega_{p}$, chosen near the readout-resonator frequency $\omega_{0}$, is used to monitor the complex transmission coefficient $S_{21}$. A second tone, at the swept spectroscopy frequency $\omega_{d}$, excites transitions of the coupled transmon–resonator system. Accordingly, the measured response can be written as $S_{21}\left(\omega_{p};\omega_{d}\right),$ where the transmission is measured at the fixed probe frequency while the spectroscopy frequency is swept. This separation between the spectroscopy and measurement tones is standard in circuit quantum electrodynamics [7,47].

Our two-tone measurements can be analyzed within the single-mode Hamiltonian introduced in Section 2.2 because the measured transitions belong to a transmon coupled to a single resonator mode. The two externally applied microwave tones serve to excite and probe transitions between the dressed eigenstates of this same coupled system, which involves a single quantized resonator mode. The bosonic mode retained in the Hamiltonian contains the complete Fock ladder n=0,1,2,…, allowing the measured transition frequencies to be related to both the single- and few-photon manifolds of the transmon–resonator system. This correspondence has direct experimental support: Fink et al. used a spectroscopic pump–probe technique to resolve “atom–photon superposition states involving up to two photons,” whereas Satrya et al. explicitly identify the possibility of performing two-tone qubit spectroscopy “in the single-photon regime” [48,49] and low-occupation on-chip coherent-field [50,51,52]. These results provide direct precedent for applying a single-mode, low-photon-number description to two-tone spectroscopic measurements.

We quantify the residual resonator loading during spectroscopic calibration from the calibrated microwave-line attenuation. For the representative device XMON2-1, the spectroscopy conditions give ${\bar{n}}_{d}≲2.4\times 10^{-5}$ at the corresponding detuned operating point. The measurement therefore probes the device in a low-occupation coherent-field regime. The complete microwave-power budget, resonator-linewidth estimate, and calculation of ${\bar{n}}_{d}$ are given in Appendix D.

#### Spectroscopic Fitting and Extracted Parameters

**Figure 10:**
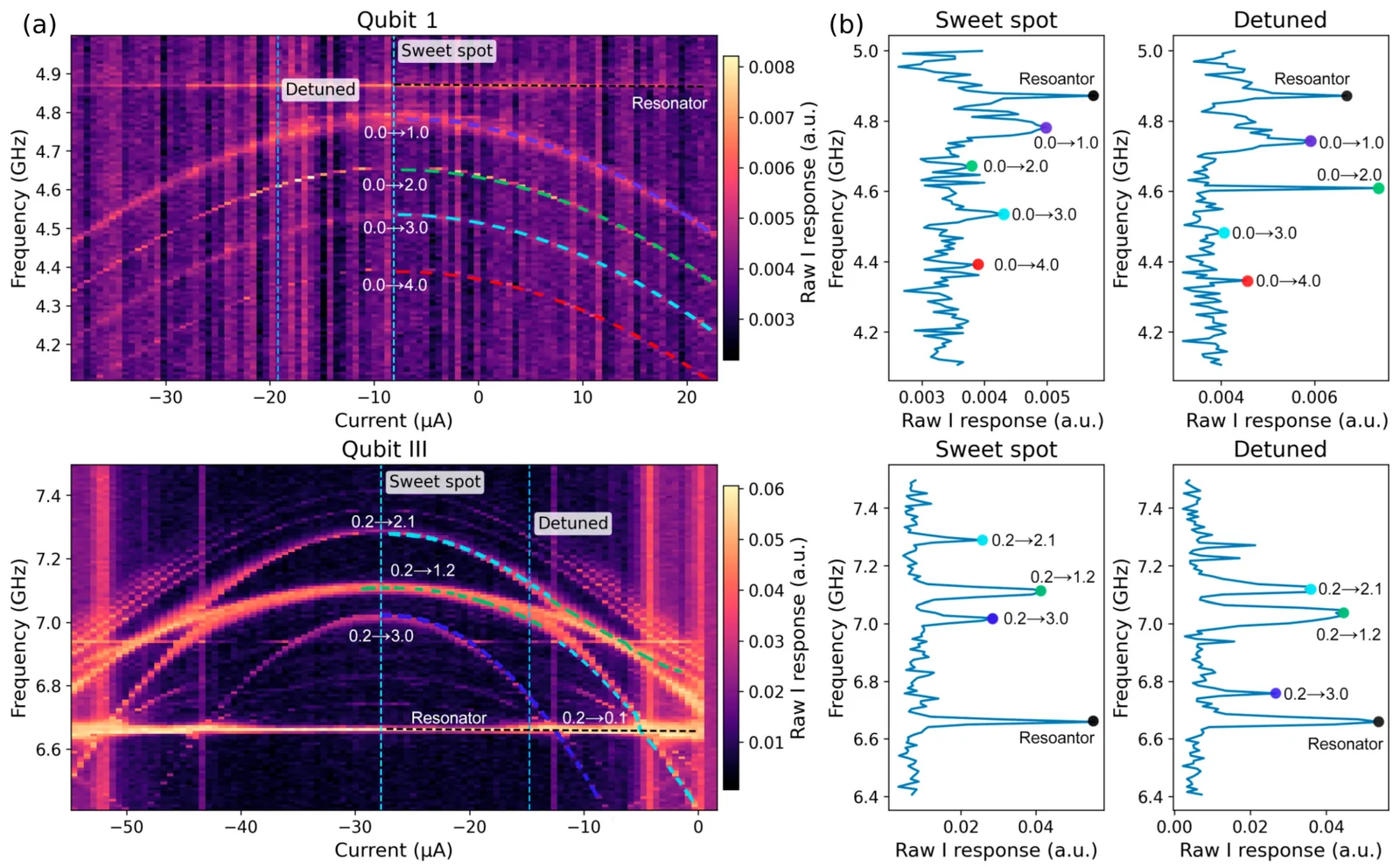
Experimentally measured two-tone spectroscopy and numerical fit for transmon–resonator systems 1 and III: (**a**) measured two-tone spectroscopy with numerically calculated transmon–resonator transitions; (**b**) $|S_{21}\left(\frac{\omega_{d}}{2\pi }\right)|$ at fixed coil current—at the sweet spot ($\Phi_{\mathrm{ext}}=0$) and detuned.

The numerical fit procedure based on the theory of Section 2.2 was carried out in several stages. First, we analyze single-tone spectroscopy to find approximate parameters of the system: the bare resonator frequency $\omega_{0}$, the transmon frequency $\omega_{01}$, the transmon–resonator coupling strength *g*, and the SQUID asymmetry *d*. Second, we calculate the transmon island capacitance using electromagnetic simulations to estimate the charging energy $E_{C}$. Finally, we employ the scqubits package as the numerical backend and QFit [53] for interactive calibration, transition extraction, and semi-automated fitting, enabling consistent extraction of $E_{J\Sigma }$, $E_{C}$, *d*, $\omega_{0}$, and *g* from two-tone spectroscopy (see Figure 10a). For each value of the coil current, we select the most visible peaks in $\left|S_{21}\left(\omega_{p};\omega_{d}\right)\right|$, as illustrated in Figure 10b. Finding such peaks for different values of the coil current traces out the transitions of the transmon–resonator system. Comparing these transitions with the numerically calculated ones enables automated parameter fitting (the typical RMSE between experimental and numerical transitions was less than 10MHz). The fitted parameters for the two datasets are summarized in Table A1 and Table A2 of Appendix B.

Across the measured samples, we obtain charging energies in the range $E_{C}=0.210$–0.301GHz, qudit–resonator coupling strengths g=0.028–0.056GHz, resonator frequencies $\omega_{0}/2\pi =6.48$–6.94GHz, and coherence times up to $T_{1}=27\;\mu s$ and $T_{2}^{*}=5.47\;\mu s$. These values calibrate the qudit ladder, the number of spectroscopically resolved levels, and the coherence window available for the detector protocol. The theoretical analysis of Section 3 uses the experimentally extracted transmon parameters as fixed input (Section 3.4) and asks which interaction regimes between the qudit and a signal mode maximize the contrast between classical and quantized fields, keeping in mind that the detuning between the resonator and transmon frequencies can be easily controlled in a wide range by applying a magnetic flux to the qudit.

## 5. Detector Protocol and Experimentally Guided Design Rules

Figure 2 and Figure 3 show that, for equal initial field energy and vanishing qudit nonlinearity, a quantized field with a fixed photon number populates only a finite set of qudit levels and follows a binomial population law, whereas a classical drive produces a broader, Poisson-like population pattern. Once the transmon nonlinearity is restored, the contrast becomes operationally stronger: Figure 4 and Figure 5 show that an appropriate detuning can selectively enhance a high-lying qudit level in the quantized case, while the corresponding population peak generated by a classical field of the same initial energy remains far weaker. Figure 6 and Figure 7 extend this comparison to a quantized coherent state of the same mean energy and show that the same operating point separates all three inputs. In particular, the fourth excited level is a natural discriminator for the $k_{0}=4$ sector. Figure 8 extends the Fock–coherent comparison to the operating point of Section 3.4 and shows distinct target-level populations $W_{4}$ at the common interaction time $t^{*}=1.8\;\mu s$.

This directly defines the detector cycle already outlined in Section 2.1. The qudit is initialized in |0〉, brought into interaction with the signal mode for a fixed time $t_{\mathrm{int}}$ at a detuning selected from the population maps of Figure 2, Figure 3, Figure 4, Figure 5, Figure 6 and Figure 7, and then flux-tuned back to the dispersive readout point, where the population vector W is measured. A nonclassical few-photon signal is identified by a narrow detuning interval in which a selected high-lying population $W_{n}$ becomes strongly enhanced; a coherent state of the same energy is recognized by the Poisson-bounded value of the same $W_{n}$ together with a non-monotonous character of population of other levels and population tail at $n>k_{0}$; a classical drive is recognized by a broad population profile extending well above $n=k_{0}$. In the linear regime, the bound of Equation (15) turns the same readout into a nonclassicality test that needs no reference input; in the nonlinear regime, the detector is operated as a discriminator among the calibrated set of inputs that the source can prepare, with the classical drive and the coherent state as the two classical-like references.

The detector therefore operates in two separately optimized stages. During the interaction stage, the qudit–signal detuning is set close to the operating point selected from the population maps, for example, near $\Delta \omega_{\mathrm{opt}}^{\left(i\right)}$ of Equation (16) when discrimination is performed via level |i〉. During the readout stage, the qudit is returned to the dispersive regime with respect to the readout resonator. This two-stage protocol combines the strong population contrast identified theoretically with the experimentally convenient dispersive measurement used in Section 4. Appendix E presents a phenomenological dissipative estimate of the interaction-stage population contrast.

Among the measured devices, the XMON2 samples are the best starting point for such a detector. In particular, samples 1 and 4 combine long coherence times with comparatively large coupling,

$$T_{1}≃27\;\mu s,\;T_{2}^{*}=5.47\;\mu s,\;g=0.046\;\mathrm{GHz}$$

and

$$T_{1}≃27\;\mu s,\;T_{2}^{*}=3.32\;\mu s,\;g=0.056\;\mathrm{GHz},$$

while sample 5 provides access to six resolved qudit levels. Accordingly, a first detector demonstrator should use one of these devices, monitor $W_{2}$, $W_{3}$ and $W_{4}$ for the $k_{0}=2,3,4$ sectors, and choose $t_{\mathrm{int}}$ such that the selected population maximum is reached well before the measured $T_{1}$ and $T_{2}^{*}$ limits.

A further design constraint follows from Equation (16): the detuning $\Delta \omega_{\mathrm{opt}}^{\left(k_{0}\right)}=\left(k_{0}+1\right)E_{C}/\left(2\hbar \right)$ is positive: the qudit must sit above the signal mode by $\left(k_{0}+1\right)E_{C}/2h$, i.e., by 545 MHz for $k_{0}=4$ and $E_{C}/h=218$ MHz. Flux tuning can only lower the transmon frequency, so this condition is met when the sweet-spot frequency $\omega_{p}\left(0\right)$ exceeds $\omega_{0}$ by at least this amount. Among the measured devices, this holds for XMON1-III, whose sweet spot lies 0.787 GHz above its resonator while its charging energy $E_{C}/h=0.301$ GHz puts $\Delta \omega_{\mathrm{opt}}^{\left(4\right)}/2\pi =0.75$ GHz within 40 MHz of the sweet spot; the XMON2 devices, with the better coherence, have their transmons below the resonator and call for a resonator redesigned ≈0.55 GHz below the sweet-spot transmon frequency. Placing the interaction point at the sweet spot is also the natural way to use the best available $T_{2}^{*}$, since the first-order sensitivity to flux noise vanishes there.

The protocol does not require the photon number to be known in advance. The detuning $\Delta \omega_{\mathrm{opt}}^{\left(n\right)}$ takes discrete values (n+1)|γ|, one per candidate sector $n=1,\ldots ,N_{lev}-1$; the flux bias is stepped through these values, and at each step, the population vector is read out at an interaction time selected from the numerical population dynamics for each detuning. A Fock state $|k_{0}\rangle$ yields a near-complete transfer at every step $n\leq k_{0}$ and none for $n>k_{0}$, because the level $n>k_{0}$ cannot be reached with $k_{0}$ photons; the highest step at which the transfer occurs identifies $k_{0}$, while the target-level population and the remaining components of the population vector provide discrimination between the calibrated input states (Table 1, Figure 8).

Two elements of the readout stage deserve a quantitative remark. (i) The flux ramp between the interaction point and the readout point must be slow with respect to the qudit anharmonicity, so that the populations follow the instantaneous levels, and fast with respect to $T_{1}$; for $E_{C}/h\approx 0.2$ GHz, ramps of 10–50 ns satisfy both conditions with a wide margin and are standard for flux-tunable transmons. As the detuning grows during the ramp, the exchange with the signal mode is switched off adiabatically, and the population vector accumulated at the interaction point is frozen; a dedicated readout resonator, as in the two-resonator layouts of Refs. [10,11], avoids crossing the signal-mode resonance during the ramp. (ii) The populations $W_{2}$–$W_{4}$ are read out by dispersive multilevel readout, for which single-shot assignment of three to five transmon levels is now routine [15,35,36,37,45,46]. With an assignment error ε per level that redistributes the population uniformly over the other levels, the measured target-level population becomes $W_{4}^{\prime }=\left(1-\varepsilon \right)W_{4}+\varepsilon \left(1-W_{4}\right)/\left(N_{lev}-1\right)$. Consequently, a difference between target-level populations is multiplied by $1-\varepsilon -\varepsilon /(N_{\mathrm{lev}}-1)$. For ε=0.1 and $N_{\mathrm{lev}}=5$, the Fock–coherent population difference at $t^{*}=1.8\;\mu s$ in Figure 8 decreases from approximately 0.72 to 0.63. For these parameters, the symmetric assignment-error model reduces the contrast without reversing the ordering of the two target-level populations.

## 6. Discussion and Conclusions

We have proposed and analyzed a few photon microwave-field detector that converts photon statistics into the level populations of a flux-tunable transmon qudit. Within the Kerr-type model, a qudit–signal detuning produces selective, periodic direct transfer of a few-photon Fock input to a higher transmon level. A coherent state of the same mean energy produces a different population response because its photon-number components evolve with different exchange frequencies. The resulting population vector provides a measurable signature for distinguishing the two inputs. The response to a maintained classical drive provides a further comparison with externally driven multilevel dynamics.

The calculations identify two uses of the population response. In the nonlinear regime, the selected-level population and the remaining components of the population vector distinguish the input states considered here. In the linear regime, Equation (15) establishes a target-population bound for every classical input state with a nonnegative Glauber–Sudarshan *P* representation. Exceeding this bound therefore witnesses nonclassicality within the linear model. Flux control sets the detuning and allows the detector response to be adapted to the photon-number sector under investigation.

Single-tone and two-tone spectroscopy, fitted using the full cosine transmon Hamiltonian, determine the circuit parameters and resolve higher transitions, while Ramsey interferometry characterizes coherence on the fundamental transition. Using the measured coupling and charging energy of device XMON2-1, g/(2π)=46MHz and $E_{C}/h=218\;\mathrm{MHz}$, the unitary calculation at the selected four-photon detuning gives $W_{4}≃0.96$ for the Fock input and $W_{4}≃0.24$ for the coherent input at the common interaction time $t^{*}=1.8\;\mu s$. This population difference of approximately 0.72 connects the detector concept to the coupling and anharmonicity scales of the fabricated devices.

These results motivate a two-stage detection protocol: interaction with the signal mode at a flux-selected detuning, followed by dispersive population readout. The signal-mode frequency and flux bias must be selected together to reach the desired multiphoton resonance. In the Kerr model, operation at the flux sweet spot requires $\omega_{p}\left(0\right)-\omega_{0}=\left(k_{0}+1\right)E_{C}/\left(2\hbar \right)$, where $\omega_{p}$ is the plasma-frequency parameter of the model. The coherence and resolved multilevel spectra of the XMON2 devices make this family a useful starting point for implementing the protocol.

A direct experimental test would prepare Fock and coherent states with the same initial mean energy and measure $W(\Phi_{\mathrm{ext}},t_{\mathrm{int}})$ across the predicted interaction settings. Calibration of multilevel readout and characterization of resonator loss and level-dependent decoherence would connect the calculated population contrast to detector performance. In this form, the transmon qudit turns from a controllable multilevel circuit-QED element into a realistic on-chip detector of nonclassical microwave-field dynamics.

## Figures and Tables

**Table 1:** Population vectors $W_{n}\left(\tau^{*}\right)$ of the three inputs at the common interaction time $\tau^{*}=\omega_{0}t^{*}=31.4$ for the operating point of Figure 4, Figure 5, Figure 6 and Figure 7 ($g=0.1\omega_{0}$, $\gamma =-0.005\omega_{0}$, $\Delta \omega =0.025\omega_{0}$). Populations below $10^{-3}$ are shown as 0.

Input	$W_{0}$	$W_{1}$	$W_{2}$	$W_{3}$	$W_{4}$	$W_{5}$	$W_{6}$	$W_{7}$	$W_{8}$	n>8
Fock, $k_{0}=4$	0	0	0.009	0	0.991	0	0	0	0	0
Coherent, $\bar{k}=4$	0.020	0.075	0.147	0.196	0.198	0.160	0.106	0.058	0.026	0.014
Classical drive	0	0.001	0.003	0.010	0.021	0.041	0.064	0.100	0.113	0.647

**Figure A2:**
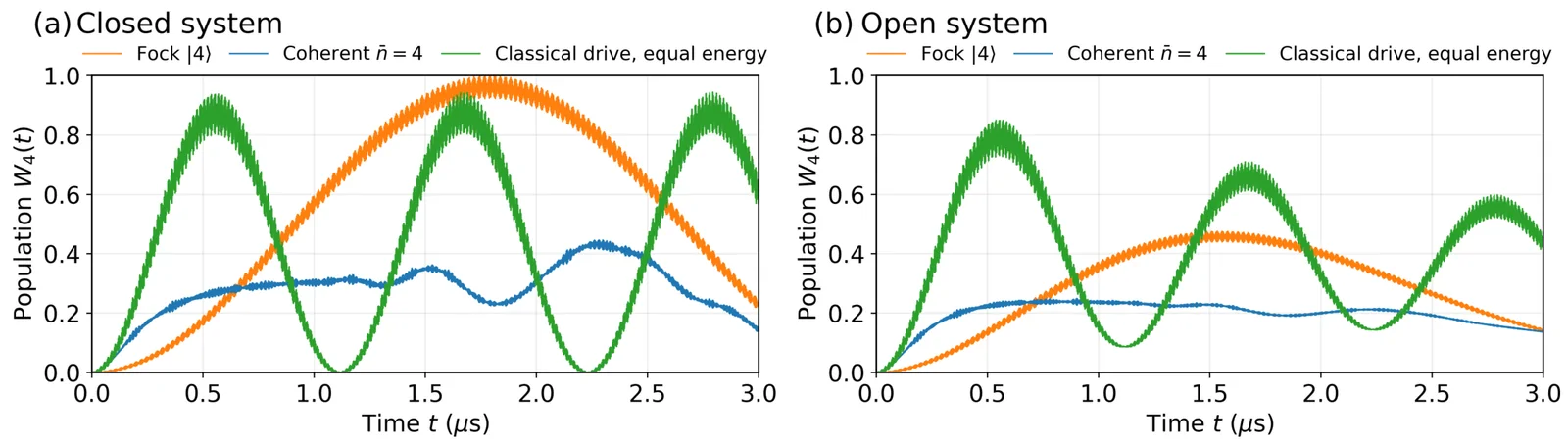
Target population $W_{4}\left(t\right)$ for a four-photon Fock input (orange), a coherent input with $\bar{k}=4$ (blue), and a maintained classical drive of equal initial energy (green). (**a**) Unitary dynamics. (**b**) Phenomenological Lindblad dynamics with $T_{1}=27\;\mu s$, assumed $T_{2}=22\;\mu s$ and κ/(2π)≃21.6kHz. Both panels use the operating point specified in the text. Resonator loss applies to the quantized inputs; the maintained classical reference includes qudit relaxation and dephasing.

## Data Availability

The data presented in this study are available on request from the corresponding author.

## Appendix A. Transmon Hamiltonian and Basis Conventions

Starting from the full transmon Hamiltonian of Equation (1), the weakly anharmonic form used in the dynamical calculations follows from the standard transmon expansion. In the regime $E_{J}\left(\Phi_{\mathrm{ext}}\right)/E_{C}\gg 1$, phase fluctuations around a minimum of the Josephson potential are small, allowing

(A1) $$\cos\hat{\varphi }\approx 1-\frac{{\hat{\varphi }}^{2}}{2}+\frac{{\hat{\varphi }}^{4}}{24}+\cdots .$$

Introducing oscillator operators $\hat{b}$ and ${\hat{b}}^{†}$ with the usual zero-point-fluctuation scaling gives, up to constants that do not affect transition frequencies, the weakly anharmonic Hamiltonian of Equation (2). The Kerr representation therefore provides the leading nonlinear approximation to the same circuit Hamiltonian that is used in the full numerical treatment.

For quantitative parameter extraction from spectroscopy, we instead diagonalize Equation (1) directly in the discrete charge basis $\left\{|n_{CP}\rangle \right\}$. In this basis,

(A2) $${\hat{H}}_{\mathrm{tr}}=\sum_{n_{CP}}4E_{C}{\left(n_{CP}-n_{g}\right)}^{2}|n_{CP}\rangle \;\langle n_{CP}|-\frac{E_{J}\left(\Phi_{\mathrm{ext}}\right)}{2}\sum_{n_{CP}}\left(|n_{CP}\rangle \;\langle n_{CP}+1|+h.c.\right),$$

which follows directly from

(A3) $$\cos\hat{\varphi }=\frac{e^{i\hat{\varphi }}+e^{-i\hat{\varphi }}}{2},\;\langle \varphi |n_{CP}\rangle =e^{i\varphi n_{CP}}.$$

The tridiagonal structure of Equation (A2) is therefore an exact representation of the cosine Hamiltonian in the charge basis rather than an additional approximation. This is the representation employed by the scqubits package used for the spectroscopic fitting procedure [54].

For capacitive transmon–resonator coupling, the interaction is proportional to the product of the transmon charge operator and a resonator voltage quadrature. The two conventions displayed in Equation (3) are related by the phase rotation $\hat{a}\to i\hat{a}$, which maps $\left(\hat{a}+{\hat{a}}^{†}\right)$ onto $i({\hat{a}}^{†}-\hat{a})$ up to an overall sign. Observable transition frequencies are invariant under this convention change provided it is applied consistently. Expanding the interaction in ladder operators produces co-rotating exchange terms and counter-rotating terms. In the usual circuit-QED regime, $g\ll \omega_{0},\omega_{01}$, the latter are neglected within the rotating-wave approximation, yielding the exchange interaction used in Section 2.3. This construction is consistent with Kerr-type multilevel transmon–resonator models used previously for few-photon dynamics [38].

To quantify the accuracy of the rotating-wave step, we have propagated the initial state ${|0\rangle }_{q}⊗{|4\rangle }_{r}$ with the full laboratory-frame Hamiltonian

(A4) $${\hat{H}}_{\mathrm{full}}=\hbar \omega_{0}{\hat{n}}_{a}+\hbar \left(\omega_{0}+\Delta \omega \right){\hat{n}}_{b}+\hbar \gamma {\hat{n}}_{b}\left({\hat{n}}_{b}+1\right)+\hbar \frac{g}{2}\left(\hat{b}+{\hat{b}}^{†}\right)\left(\hat{a}+{\hat{a}}^{†}\right),$$

which differs from Equation (4) by the counter-rotating terms $\hat{b}\hat{a}$ and ${\hat{b}}^{†}{\hat{a}}^{†}$, and compared the qudit populations with those of the RWA model. In the strong coupling regime (Figure A1a), a relatively small correction is observed, whereas the nonlinearity-dominated regime (Figure A1b) is more sensitive to additional detunings due to g<<γ. As a result, the narrow resonance shifted quite significantly in frequency (i.e., the theoretical optimal detuning, which is −5γ, in the case of a transition from Kerr nonlinearity to ${\left(\hat{b}+{\hat{b}}^{†}\right)}^{4}$ nonlinearity in the mode of prevailing nonlinearity ($\frac{\left|\gamma \right|}{g}=2.37$) has ceased to be optimal). At the strong-coupling point, the counter-rotating terms lower the first maximum of $W_{4}$ from 0.991 to 0.982 and shift it from τ=31.4 to τ=30.8; the subsequent maxima follow the RWA result with the same accuracy. At the measured device parameters (Section 3.4), the two calculations differ by less than $5\times 10^{-3}$ over the whole interaction window. The Bloch–Siegert-type corrections of order $g^{2}/\omega_{0}$ thus slightly renormalize the optimal detuning and leave the selective transfer intact.

## Appendix B. Two-Tone Spectroscopy Fitting Results and Experimentally Measured Coherence Times

Values in GHz are reported in frequency units (commonly E/h, ω/2π). The $N_{lev}$ parameter indicates the number of transmon energy levels that were found using two-tone spectroscopy and numerical fitting.

**Table A1:** Two-tone spectroscopy fitting results and experimentally measured coherence times for dataset XMON1.

Parameter	Qudit №				
	I	II	III	IV	V
$E_{J\Sigma }$ (GHz)	23.634	22.419	23.013	22.791	22.366
$E_{C}$ (GHz)	0.211	0.216	0.301	0.211	0.220
*d* (a.u.)	0.648	0.317	0.345	0.535	0.458
$\omega_{p}\left(\Phi_{\mathrm{ext}}=0\right)$ (GHz)	6.316	6.224	7.444	6.203	6.274
$\omega_{0}$ (GHz)	6.479	6.572	6.657	6.755	6.936
$\Delta \omega (\Phi_{\mathrm{ext}}=0)$ (GHz)	−0.163	−0.348	0.787	−0.552	−0.662
*g* (GHz)	0.030	0.036	0.029	0.032	0.035
$N_{lev}$	5	4	4	3	3
$T_{1}$ (μs)	3.7	4.3	2.6	6.03	2.5
$T_{2}^{*}$ (μs)	0.27	1.00	0.75	1.14	0.61

**Table A2:** Two-tone spectroscopy fitting results and experimentally measured coherence times for dataset XMON2.

Parameter	Qudit №				
	1	2	3	4	5
$E_{J\Sigma }$ (GHz)	14.406	14.753	17.810	13.775	17.534
$E_{C}$ (GHz)	0.218	0.215	0.212	0.215	0.210
*d* (a.u.)	0.674	0.817	0.609	0.381	0.598
$\omega_{p}\left(\Phi_{\mathrm{ext}}=0\right)$ (GHz)	5.012	5.037	5.496	4.868	5.427
$\omega_{0}$ (GHz)	6.494	6.612	6.706	6.802	6.895
$\Delta \omega (\Phi_{\mathrm{ext}}=0)$ (GHz)	−1.482	−1.575	−1.210	−1.934	−1.467
*g* (GHz)	0.046	0.035	0.028	0.056	0.047
$N_{lev}$	5	5	5	5	6
$T_{1}$ (μs)	27	9	19	27	21
$T_{2}^{*}$ (μs)	5.47	2.45	3.03	3.32	1.45

## Appendix C. Experimental Details

The device under study is a standard cQED chip comprising a high-quality-factor ($Q∼10^{4}$–$10^{5}$) half-wavelength coplanar waveguide resonator with resonance frequencies $\omega_{0}/2\pi =6.48$–6.94GHz capacitively coupled to a tunable transmon qudit, with Josephson plasma frequencies $\omega_{p}/2\pi \approx 4.87$–7.44GHz at the sweet spot $\Phi_{\mathrm{ext}}=0$. The chip is mounted in a copper sample box and cooled below 17mK in a wet dilution refrigerator [55]. The resonator and the large-scale transmon structures are fabricated from thin-film aluminium, the Josephson tunnel junction is realized using a standard $\mathrm{Al}/{\mathrm{AlO}}_{x}/\mathrm{Al}$ stack [56]. Microwave control pulses for the Ramsey experiment are generated using single-sideband mixing with local oscillators and arbitrary waveform generators (AWGs). Along with the continuous microwave tone applied to generate the on-chip field, these signals are attenuated at successive cryostat stages before reaching the sample. Dispersive readout of the qudit state is performed via the resonator [43], and the resulting output signal is measured by a vector network analyzer (VNA) and processed by the measurement software [57].

## Appendix D. Microwave Powers and Resonator Occupation

The room-temperature power of the swept spectroscopy tone was

$$P_{d,\mathrm{src}}=-40\;\mathrm{dBm}.$$

The total estimated attenuation between the microwave generator and the sample was approximately

$$A_{d}=63\;\mathrm{dB},$$

including the cryogenic attenuators and the insertion losses of the microwave line. The corresponding incident spectroscopy-tone power at the device was therefore

$$P_{d,\mathrm{chip}}=P_{d,\mathrm{src}}-A_{d}≃-103\;\mathrm{dBm}=5.0\times 10^{-14}\;W.$$

Here, 63dB is the total line attenuation, whereas −103dBm is the estimated absolute power incident on the device.

The resonator occupation induced by the spectroscopy tone is

(A5) $${\bar{n}}_{d}=\frac{\kappa_{\mathrm{ext}}}{{\left(\kappa /2\right)}^{2}+\Delta_{\mathrm{dr}}^{2}}\frac{P_{d,\mathrm{chip}}}{\hbar \omega_{d}},\;\Delta_{\mathrm{dr}}=\omega_{d}-\omega_{0},$$

where κ is the total resonator energy-decay rate and $\kappa_{\mathrm{ext}}$ is the coupling rate to the driven port [7,47].

As a representative example, for device XMON2-1 at its sweet spot, the experimentally determined parameters are

$$Q_{l}=3.0\times 10^{5},\;\frac{\omega_{0}}{2\pi }=6.494\;\mathrm{GHz},\;\frac{|\Delta_{\mathrm{dr}}|}{2\pi }=1.482\;\mathrm{GHz}.$$

The corresponding resonator linewidth is

(A6) $$\frac{\kappa }{2\pi }=\frac{\omega_{0}/2\pi }{Q_{l}}≃21.6\;\mathrm{kHz}.$$

Using the conservative bound $\kappa_{\mathrm{ext}}\leq \kappa$ in Equation (A5), we obtain

(A7) $${\bar{n}}_{d}≲2.4\times 10^{-5}\ll 1.$$

Thus, at this detuned operating point, the spectroscopy tone produces only weak off-resonant loading of the resonator. A coherent microwave tone with $\bar{n}<1$ nevertheless remains a coherent state with Poissonian photon-number statistics. The estimate above therefore characterizes the low-occupation coherent-field regime of the spectroscopic calibration.

## Appendix E. Closed Versus Open Quantum System Dynamics: A Time-Scale Perspective

We supplement the unitary results of Section 3.4 with a phenomenological Lindblad calculation [58,59] including qudit relaxation, pure dephasing and resonator loss (Figure A2). The quantized inputs evolve under the Hamiltonian of Equation (4), using the measured XMON2-1 coupling and charging energy, g/(2π)=46MHz and $E_{C}/h=218\;\mathrm{MHz}$, with $\omega_{0}/\left(2\pi \right)=6.494\;\mathrm{GHz}$ and the selected detuning Δω/(2π)=545MHz. The dissipative parameters are $T_{1}=27\;\mu s$, $Q_{l}=3\times 10^{5}$ and κ/(2π)≃21.6kHz. The coherence time $T_{2}=22\;\mu s$ is assumed.

For equal-prior Fock and coherent inputs with $k_{0}=\bar{k}=4$, and an ideal binary readout distinguishing ${|4\rangle }_{q}$ from all other qudit levels, the target-population contrast and the corresponding minimum decision error are

(A8) $$\begin{array}{cc} C_{4}\left(t\right) & =W_{4}^{F}\left(t\right)-W_{4}^{\mathrm{coh}}\left(t\right),\\ P_{\mathrm{err}}^{\left(4\right)}\left(t\right) & =\frac{1-|C_{4}\left(t\right)|}{2}. \end{array}$$

At the common interaction time $t^{*}=1.8\;\mu s$ used in Figure 8, approximate readings of the dissipative curves give $W_{4}^{F}≃0.44$ and $W_{4}^{\mathrm{coh}}≃0.20$, compared with the unitary values 0.96 and 0.24. Dissipation therefore reduces $C_{4}$ from approximately 0.72 to 0.24 and increases $P_{\mathrm{err}}^{\left(4\right)}$ from approximately 0.14 to 0.38.

## References

[1] Vlastakis B., Kirchmair G., Leghtas Z., Nigg S.E., Frunzio L., Girvin S.M., Mirrahimi M., Devoret M.H., Schoelkopf R.J. (2013). Deterministically encoding quantum information using 100-photon Schrödinger cat states. Science.

[2] Leghtas Z., Touzard S., Pop I.M., Kou A., Vlastakis B., Petrenko A., Sliwa K.M., Narla A., Shankar S., Hatridge M.J. (2015). Confining the state of light to a quantum manifold by engineered two-photon loss. Science.

[3] Wang C., Gao Y.Y., Reinhold P., Heeres R.W., Ofek N., Chou K., Axline C., Reagor M., Blumoff J., Sliwa K.M. (2016). A Schrödinger cat living in two boxes. Science.

[4] Ofek N., Petrenko A., Heeres R., Reinhold P., Leghtas Z., Vlastakis B., Liu Y., Frunzio L., Girvin S.M., Jiang L. (2016). Extending the lifetime of a quantum bit with error correction in superconducting circuits. Nature.

[5] Kwon S., Hoshi D., Nagase T., Sugiyama D., Mukai H., Takemura K., Kojima R., Zhou Y., Watabe S., Yoshihara F. (2026). Realisation of Protected Cat Qutrit via Engineered Quantum Tunnelling. arXiv.

[6] Gu X., Kockum A.F., Miranowicz A., Liu Y.x., Nori F. (2017). Microwave photonics with superconducting quantum circuits. Phys. Rep..

[7] Blais A., Grimsmo A.L., Girvin S.M., Wallraff A. (2021). Circuit quantum electrodynamics. Rev. Mod. Phys..

[8] Zakharov R.V., Tikhonova O.V., Klenov N.V., Soloviev I.I., Antonov V.N., Yakovlev D.S. (2024). Solid-State Qubit as an On-Chip Controller for Non-Classical Field States. Adv. Quantum Technol..

[9] Devoret M., Girvin S., Schoelkopf R. (2007). Circuit-QED: How strong can the coupling between a Josephson junction atom and a transmission line resonator be?. Ann. Der Phys..

[10] Hofheinz M., Weig E.M., Ansmann M., Bialczak R.C., Lucero E., Neeley M., O’Connell A.D., Wang H., Martinis J.M., Cleland A.N. (2008). Generation of Fock states in a superconducting quantum circuit. Nature.

[11] Hofheinz M., Wang H., Ansmann M., Bialczak R.C., Lucero E., Neeley M., O’Connell A.D., Sank D., Wenner J., Martinis J.M. (2009). Synthesizing arbitrary quantum states in a superconducting resonator. Nature.

[12] Kirchmair G., Vlastakis B., Leghtas Z., Nigg S., Paik H., Ginossar E., Mirrahimi M., Frunzio L., Girvin S., Schoelkopf R. (2013). Observation of quantum state collapse and revival due to the single-photon Kerr effect. Nature.

[13] Peng Z., De Graaf S., Tsai J., Astafiev O. (2016). Tuneable on-demand single-photon source in the microwave range. Nat. Commun..

[14] Zhao Y.J., Wang C., Zhu X., Liu Y.x. (2016). Engineering entangled microwave photon states through multiphoton interactions between two cavity fields and a superconducting qubit. Sci. Rep..

[15] Jerger M., Macha P., Hamann A.R., Reshitnyk Y., Juliusson K., Fedorov A. (2016). Realization of a Binary-Outcome Projection Measurement of a Three-Level Superconducting Quantum System. Phys. Rev. Appl..

[16] Dmitriev A.Y., Shaikhaidarov R., Antonov V., Hönigl-Decrinis T., Astafiev O. (2017). Quantum wave mixing and visualisation of coherent and superposed photonic states in a waveguide. Nat. Commun..

[17] Dmitriev A.Y., Shaikhaidarov R., Hönigl-Decrinis T., De Graaf S., Antonov V., Astafiev O. (2019). Probing photon statistics of coherent states by continuous wave mixing on a two-level system. Phys. Rev. A.

[18] Hajr A., Qing B., Wang K., Koolstra G., Pedramrazi Z., Kang Z., Chen L., Nguyen L.B., Jünger C., Goss N. (2024). High-Coherence Kerr-Cat Qubit in 2D Architecture. Phys. Rev. X.

[19] Li D.S., Kang Y.H., Shi Z.C., Xiao Y., Chen Y.H., Xia Y. (2026). Efficient and flexible preparation of photonic NOON states in a superconducting system. Phys. Rev. A.

[20] Mallet F., Castellanos-Beltran M.A., Ku H.S., Glancy S., Knill E., Irwin K.D., Hilton G.C., Vale L.R., Lehnert K.W. (2011). Quantum state tomography of an itinerant squeezed microwave field. Phys. Rev. Lett..

[21] Eichler C., Bozyigit D., Lang C., Steffen L., Fink J., Wallraff A. (2011). Experimental state tomography of itinerant single microwave photons. Phys. Rev. Lett..

[22] Johansson J., Saito S., Meno T., Nakano H., Ueda M., Semba K., Takayanagi H. (2006). Vacuum Rabi oscillations in a macroscopic superconducting qubit LC oscillator system. Phys. Rev. Lett..

[23] Schuster D.I., Houck A.A., Schreier J., Wallraff A., Gambetta J.M., Blais A., Frunzio L., Majer J., Johnson B., Devoret M.H. (2007). Resolving photon number states in a superconducting circuit. Nature.

[24] Johnson B.R., Reed M.D., Houck A.A., Schuster D.I., Bishop L.S., Ginossar E., Gambetta J.M., DiCarlo L., Frunzio L., Girvin S.M. (2010). Quantum non-demolition detection of single microwave photons in a circuit. Nat. Phys..

[25] Sun L., Petrenko A., Leghtas Z., Vlastakis B., Kirchmair G., Sliwa K.M., Narla A., Hatridge M., Shankar S., Blumoff J. (2014). Tracking photon jumps with repeated quantum non-demolition parity measurements. Nature.

[26] Inomata K., Lin Z., Koshino K., Oliver W.D., Tsai J.S., Yamamoto T., Nakamura Y. (2016). Single microwave-photon detector using an artificial *Λ*-type three-level system. Nat. Commun..

[27] Besse J.C., Gasparinetti S., Collodo M.C., Walter T., Kurpiers P., Pechal M., Eichler C., Wallraff A. (2018). Single-shot quantum nondemolition detection of individual itinerant microwave photons. Phys. Rev. X.

[28] Kono S., Koshino K., Tabuchi Y., Noguchi A., Nakamura Y. (2018). Quantum non-demolition detection of an itinerant microwave photon. Nat. Phys..

[29] Koch J., Yu T.M., Gambetta J., Houck A.A., Schuster D.I., Majer J., Blais A., Devoret M.H., Girvin S.M., Schoelkopf R.J. (2007). Charge-insensitive qubit design derived from the Cooper pair box. Phys. Rev. A.

[30] Peterer M.J., Bader S.J., Jin X., Yan F., Kamal A., Gudmundsen T.J., Leek P.J., Orlando T.P., Oliver W.D., Gustavsson S. (2015). Coherence and decay of higher energy levels of a superconducting transmon qubit. Phys. Rev. Lett..

[31] Shapiro D.S. (2021). Transport of pseudothermal photons through an anharmonic cavity. Sci. Rep..

[32] Bishop L.S., Chow J., Koch J., Houck A., Devoret M., Thuneberg E., Girvin S., Schoelkopf R. (2009). Nonlinear response of the vacuum Rabi resonance. Nat. Phys..

[33] Deppe F., Mariantoni M., Menzel E., Marx A., Saito S., Kakuyanagi K., Tanaka H., Meno T., Semba K., Takayanagi H. (2008). Two-photon probe of the Jaynes–Cummings model and controlled symmetry breaking in circuit QED. Nat. Phys..

[34] Popolitova D.V., Klenov N.V., Soloviev I.I., Bakurskiy S.V., Tikhonova O.V. (2019). Unipolar magnetic field pulses as an advantageous tool for ultrafast operations in superconducting Josephson “atoms”. Beilstein J. Nanotechnol..

[35] Bianchetti R., Filipp S., Baur M., Fink J.M., Lang C., Steffen L., Boissonneault M., Blais A., Wallraff A. (2010). Control and tomography of a three level superconducting artificial atom. Phys. Rev. Lett..

[36] Kristen M., Schneider A., Stehli A., Wolz T., Danilin S., Ku H.S., Long J., Wu X., Lake R., Pappas D.P. (2020). Amplitude and frequency sensing of microwave fields with a superconducting transmon qudit. npj Quantum Inf..

[37] Wang Z., Parker R.W., Champion E., Blok M.S. (2025). High-EJ/EC transmon qudits with up to 12 levels. Phys. Rev. Appl..

[38] Solovykh I.A., Pashchenko A.V., Maleeva N.A., Klenov N.V., Tikhonova O.V., Soloviev I.I. (2025). Few-photon microwave fields for superconducting transmon-based qudit control. Beilstein J. Nanotechnol..

[39] Pogosov W.V., Dmitriev A.Y., Astafiev O.V. (2021). Effects of photon statistics in wave mixing on a single qubit. Phys. Rev. A.

[40] Gunin S.A., Dmitriev A.Y., Vasenin A.V., Tikhonov K.S., Fedorov G.P., Astafiev O.V. (2023). Quantum and classical field scattered on a single two-level system. Phys. Rev. A.

[41] Dmitriev A.Y., Vasenin A.V., Gunin S.A., Remizov S.V., Elistratov A.A., Pogosov W.V., Astafiev O.V. (2025). Direct experimental observation of sub-Poissonian photon statistics by means of multiphoton scattering on a two-level system. Phys. Rev. A.

[42] Dumas M.F., Groleau-Paré B., McDonald A., Muñoz Arias M.H., Lledó C., D’Anjou B., Blais A. (2024). Measurement-Induced Transmon Ionization. Phys. Rev. X.

[43] Fedorov G., Yursa V., Efimov A., Shiianov K., Dmitriev A.Y., Rodionov I., Dobronosova A., Moskalev D., Pishchimova A., Malevannaya E. (2020). Light dressing of a diatomic superconducting artificial molecule. Phys. Rev. A.

[44] Fedorov G.P., Remizov S., Shapiro D., Pogosov W., Egorova E., Tsitsilin I., Andronik M., Dobronosova A., Rodionov I., Astafiev O. (2021). Photon transport in a Bose-Hubbard chain of superconducting artificial atoms. Phys. Rev. Lett..

[45] Goss N., Ferracin S., Hashim A., Carignan-Dugas A., Kreikebaum J.M., Naik R.K., Santiago D.I., Siddiqi I. (2024). Extending the computational reach of a superconducting qutrit processor. npj Quantum Inf..

[46] Nguyen L.B., Goss N., Siva K., Kim Y., Younis E., Qing B., Hashim A., Santiago D.I., Siddiqi I. (2024). Empowering a qudit-based quantum processor by traversing the dual bosonic ladder. Nat. Commun..

[47] Wallraff A., Schuster D., Blais A., Gambetta J., Schreier J., Frunzio L., Devoret M., Girvin S., Schoelkopf R. (2007). Sideband transitions and two-tone spectroscopy of a superconducting qubit strongly coupled to an on-chip cavity. Phys. Rev. Lett..

[48] Fink J., Göppl M., Baur M., Bianchetti R., Leek P.J., Blais A., Wallraff A. (2008). Climbing the Jaynes–Cummings ladder and observing its nonlinearity in a cavity QED system. Nature.

[49] Satrya C.D., Chang Y.C., Strelnikov A.S., Upadhyay R., Mäkinen I.K., Peltonen J.T., Karimi B., Pekola J.P. (2025). Thermal spectrometer for superconducting circuits. Nat. Commun..

[50] Yan C., Hassel J., Vesterinen V., Zhang J., Ikonen J., Grönberg L., Goetz J., Möttönen M. (2021). A low-noise on-chip coherent microwave source. Nat. Electron..

[51] Khrenov M., Yusupov R., Gubochkin G., Vozhakov V., Nazhestkin I., Belanovsky A., Ionin A., Gladilovich P., Khismatullin G., Filippenko L. (2026). Tunable Josephson Generator for Qubit Excitation and Two-Tone Spectroscopy. Adv. Quantum Technol..

[52] Vervoort S., Nulens L., Chaves D.A., Dausy H., Reniers S., Abouelela M., Cools I.P., Silhanek A.V., Van Bael M.J., Raes B. (2025). DC-operated Josephson junction arrays as a cryogenic on-chip microwave measurement platform. Commun. Phys..

[53] Zhao T., Chen D., Koch J. (2025). QFit: Interactive Parameter Fitting for Superconducting Circuits. GitHub Repository (Beta). https://github.com/scqubits/qfit.

[54] Groszkowski P., Koch J. (2021). Scqubits: A Python package for superconducting qubits. Quantum.

[55] Nazhestkin I.A., Gubochkin G.I., Shvartzberg J., Rajam S.P.M., Egorov S.V., Gurtovoi V.L., Ryazanov V.V., Stolyarov V.S., Yakovlev D.S. (2025). Enhancing Signal Purity in Josephson Structure Measurements. Adv. Phys. Res..

[56] Smirnov N.S., Krivko E.A., Solovyova A.A., Ivanov A.I., Rodionov I.A. (2024). Wiring surface loss of a superconducting transmon qubit. Sci. Rep..

[57] Fedorov G., Ustinov A. (2019). Automated analysis of single-tone spectroscopic data for cQED systems. Quantum Sci. Technol..

[58] Breuer H.P., Petruccione F. (2002). The Theory of Open Quantum Systems.

[59] Lindblad G. (1976). On the generators of quantum dynamical semigroups. Commun. Math. Phys..

